# A chromosome-level reference genome of non-heading Chinese cabbage [*Brassica campestris* (syn*. Brassica rapa*) ssp*. chinensis*]

**DOI:** 10.1038/s41438-020-00449-z

**Published:** 2020-12-28

**Authors:** Ying Li, Gao-Feng Liu, Li-Ming Ma, Tong-Kun Liu, Chang-Wei Zhang, Dong Xiao, Hong-Kun Zheng, Fei Chen, Xi-Lin Hou

**Affiliations:** 1 State Key Laboratory of Crop Genetics & Germplasm Enhancement, Key Laboratory of Biology and Genetic Improvement of Horticultural Crops (East China), Ministry of Agriculture and Rural Affairs of the P. R. China, Engineering Research Center of Germplasm Enhancement and Utilization of Horticultural Crop, Ministry of Education of the P. R. China, College of Horticulture, Nanjing Agricultural University, Nanjing, 210095 China; 2 Biomarker Technologies Corporation, Beijing, 101300 China

**Keywords:** Comparative genomics, Genome

## Abstract

Non-heading Chinese cabbage (NHCC) is an important leafy vegetable cultivated worldwide. Here, we report the first high-quality, chromosome-level genome of NHCC001 based on PacBio, Hi-C, and Illumina sequencing data. The assembled NHCC001 genome is 405.33 Mb in size with a contig N50 of 2.83 Mb and a scaffold N50 of 38.13 Mb. Approximately 53% of the assembled genome is composed of repetitive sequences, among which long terminal repeats (LTRs, 20.42% of the genome) are the most abundant. Using Hi-C data, 97.9% (396.83 Mb) of the sequences were assigned to 10 pseudochromosomes. Genome assessment showed that this *B. rapa* NHCC001 genome assembly is of better quality than other currently available *B. rapa* assemblies and that it contains 48,158 protein-coding genes, 99.56% of which are annotated in at least one functional database. Comparative genomic analysis confirmed that *B. rapa* NHCC001 underwent a whole-genome triplication (WGT) event shared with other *Brassica* species that occurred after the WGD events shared with *Arabidopsis*. Genes related to ascorbic acid metabolism showed little variation among the three *B. rapa* subspecies. The numbers of genes involved in glucosinolate biosynthesis and catabolism were higher in NHCC001 than in Chiifu and Z1, due primarily to tandem duplication. The newly assembled genome will provide an important resource for research on *B. rapa*, especially *B. rapa* ssp. *chinensis*.

## Introduction

The *Brassica* genus comprises various economically important crops, many of which are extensively cultivated worldwide as oil crops and leafy vegetables. Six *Brassica* crop species comprise the “U’s triangle”, which includes the three basic diploid species *B. rapa* (A genome), *B. nigra* (B genome), and *B. oleracea* (C genome), as well as the three amphidiploid species *B. juncea* (A and B genomes), *B. napus* (A and C genomes), and *B. carinata* (B and C genomes)^1^. *Brassica campestris*, which is often used as a synonym for *B. rapa*, is an agronomically important species that includes various widely cultivated subspecies such as the turnip (ssp. *rapa*), Chinese cabbage (ssp. *pekinensis*), non-heading Chinese cabbage (ssp. *chinensis*), rapini (ssp. *sylvestris*), yellow sarson types (ssp. *trilocularis*), and oil types (ssp. *oleifera*)^2,3^. *Brassica campestris* has been cultivated for specific phenotypic characteristics, such as enlarged edible roots, mid-ribs, leaves, and oil seeds. Non-heading Chinese cabbage (NHCC) is an important *B. campestris* (syn. *B. rapa*) subspecies that includes pak-choi (var. *communis* Tesn et Lee), Tacai (var. *rosularis* Tsen et Lee), Caitai (var. *tsai-tai* Hort.), Fenniecai (var. *multiceps* Hort.), and Taicai (var. *tai-tsai* Hort.). Pak-choi can be further divided into white petiole and green petiole types. It is one of the most popular vegetables in China, Vietnam, the Philippines, and other East-Asian regions and is becoming increasingly popular around the world for its sweet, succulent, and nutritious leaves and stalks.

The genomes of two model plants, the dicot *Arabidopsis* and the monocot rice, were completed in 2000 and 2002 using early generation sequencing systems^4,5^. To date, approximately 200 plant genomes have now been published^6^. In recent years, sequencing technologies have undergone tremendous development. Single-molecule sequencing, also referred to as third-generation sequencing, aims to meet the demand for high-quality plant genome assembly^7^, and PacBio and Oxford Nanopore Technology (ONT) sequencing have been used to assemble new, high-quality reference genomes for maize and tomato^8,9^. The *Brassica* genus provides a good opportunity to study genome evolution in polyploids. The first *B. rapa* genome draft published in 2011 was assembled using a whole-genome shotgun strategy with Illumina short reads^10^. The recently released *B. rapa* Chiifu genome v3.0 based on PacBio sequencing lacked nearly 20% of the expected genome content (353.14 of 442.9 Mb), and the assembly was highly fragmented (contig N50 1.45 Mb)^11^. The genome of a new morphotype, *B. rapa* Z1, was also assembled using Nanopore sequencing with a contig N50 of 5.51 Mb^12^. Previously released *B. rapa* genomes provide great convenience for both genetic and comparative genomic studies, but they cannot fully satisfy the requirements of subsequent functional genomics research and the molecular breeding of non-heading Chinese cabbage. Therefore, it is necessary to assemble a high-quality reference genome for NHCC. In addition, current sequencing technologies are evolving rapidly, and the development of improved technologies enables the production of higher quality genomes.

Here, we present a chromosome-level assembly of *B. rapa* NHCC001 produced using a combination of PacBio sequencing and chromosome conformation capture (Hi-C) technologies. Our newly assembled *B. rapa* genome achieves a high level of continuity and completeness. It provides insights into the evolution of *Brassica* and constitutes an important resource for research, especially on the molecular mechanisms that underlie agricultural traits and on the breeding of *B. rapa* ssp. *chinensis*.

## Results

### De novo genome assembly

The size of the *B. rapa* NHCC001 genome estimated by *k*-mer analysis was 477.76 Mb, larger than that of the *B. rapa* Chiifu genome, which was estimated to be 442.90 Mb (Supplementary Figure S1). The *B. rapa* NHCC001 genome heterozygosity rate was predicted to be about 0.17%, its repeat sequence content about 58.57%, and its GC content about 38.16%. We performed the genome assembly of *B. rapa* NHCC001 using 5,387,116 high-quality PacBio sequencing reads (61.44 Gb) with an N50 of 16,938 bases. Details of the PacBio sequencing reads are provided in Supplementary Table S1. All reads were assembled by SMARTdenovo into an initial genome of 384.71 Mb, which represented 80.52% of the estimated *B. rapa* NHCC001 genome size. The assembled genome contained 891 contigs with a contig N50 of 2.13 Mb and a maximum contig length of 16.11 Mb.

**Fig. 1:**
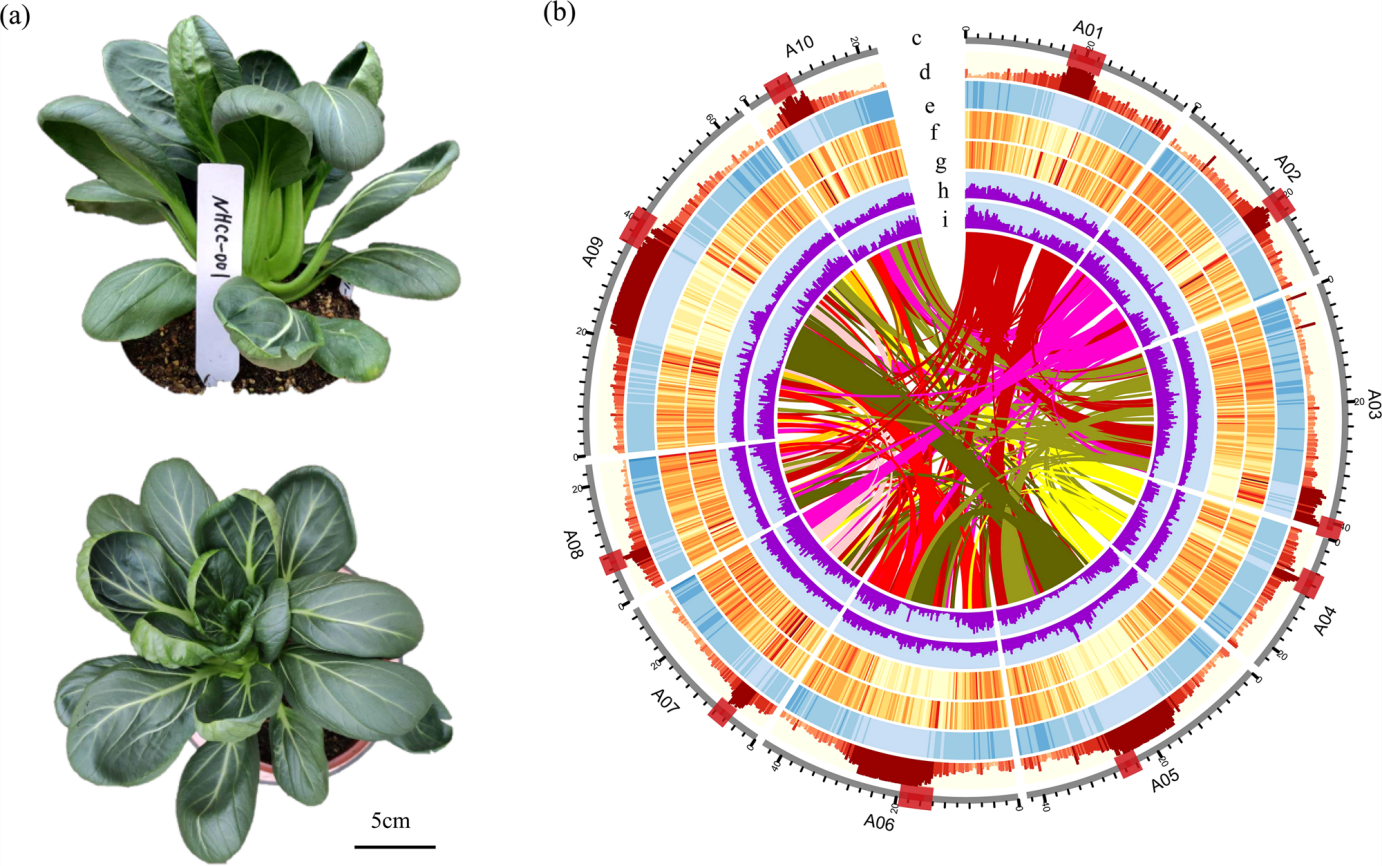
Features of NHCC001 (*Brassica campestris* (syn. *Brassica rapa*) ssp. *chinensis*) and Circos diagram of different elements on the *B. rapa* NHCC001 chromosomes. **a** A side view (top) and overhead view (bottom) of NHCC001. **b** Circos diagram. **c** Pseudomolecules with centromeres shown as red blocks. **d** Repeat density. **e** Gene density. **f** SNPs identified between *B. rapa* NHCC001 and *B. rapa* Z1. **g** SNPs identified between *B. rapa* NHCC001 and *B. rapa* Chiifu. **h** Indels identified between *B. rapa* NHCC001 and *B. rapa* Z1. **i** Indels identified between *B. rapa* NHCC001 and *B. rapa* Chiifu. Each inner curved line represents a syntenic block

Hi-C data were used to assign the resulting contigs to their chromosomal positions. We generated 68.69 Gb of clean data with ~143× coverage and anchored the assembled contigs to ten pseudochromosomes using the Hi-C data. The final chromosome-scale genome was composed of ten clusters, as indicated in the Hi-C interaction heat map (Supplementary Fig. S2). The anchored 97.90% (396.83 Mb) of assembled genome content included 553 contigs clustered by Hi-C data (Fig. 1, Supplementary Table S2). Among these clustered contigs, 300 (363.76 Mb) were anchored with defined order and orientation (Supplementary Table S2). The gap-closing step for pseudochromosomes was performed using error-corrected Nanopore clean reads (Supplementary Table S1). The final chromosome-scale genome was 405.33 Mb in length with 602 contigs (contig N50 = 2.83 Mb) and contained 312 scaffolds with a scaffold N50 of 38.13 Mb and 290 gaps (Table 1). The final genome assembly represented 84.83% of the estimated *B. rapa* NHCC001 genome, compared with 79.73% and 74.20% for the recently released Chiifu v3.0 and Z1 genomes, respectively. The GC content of the assembled genome was 37.13%, similar to the 38.16% estimate from *k*-mer analysis (Table 1).

**Table 1:** Statistics for the de novo assembled NHCC001 genome and other current *B. rapa* genome versions

	*B. rapa* (ssp*. chinensis*)	*B. rapa* (ssp. *pekinensis*)	*B. rapa* (ssp. *trilocularis*)
Accession	NHCC001	Chiifu (v3.0)	Z1
Estimated genome size (Mb)	477.76	442.90	529.00
Genome sequence size (Mb)	405.33	353.14	392.50
Contig number	602	1498	627
Contig N50 (Mb)	2.83	1.45	5.51
Contig max (Mb)	22.49	9.4	22.12
Scaffold number	312	1301	335
Scaffold N50 (Mb)	38.13	4.44	15.38
GC content (%)	37.13	36.83	37.20
Gaps total length (kb)	13,382	2,078	32,966
Annotated gene number	48,158	46,250	46,721

### Completeness and accuracy of the assembly

BUSCO v4.0.6 analysis revealed that 99.07% (1559 of 1614) of the core eukaryotic genes—including 1361 single-copy orthologs and 238 duplicated orthologs—were present in our assembly (Supplementary Table S3). The base error percentage of the genome assembly was estimated to be 0.0011% (Supplementary Table S4). More than 99.96% of the full-length transcripts assembled de novo from transcriptome data had best hits on single contigs, confirming the completeness of the genome (Supplementary Table S5). In addition, 89.05–91.19% of the clean reads from four RNA-seq libraries could be uniquely mapped to the genome (Supplementary Table S6). Taken together, these independent assessments confirmed that the *B. rapa* NHCC001 genome had high contiguity, completeness, and base accuracy.

### Genome annotation

A combination of de novo, homology-based, and transcriptome-based predictions indicated that 102.05 Mb (25.18%) of the 405.33 Mb assembled genome encoded 48,158 genes with a mean exon number of 5.08 per gene, similar to *A. thaliana* and *B. rapa*. The average gene length was 2118.99 bp, and the average exon and intron lengths were 1236.80 and 873.19 bp per gene, respectively. Among the 48,158 predicted genes, 47,872 (99.41%) could be annotated using at least one functional protein database (Supplementary Table S7). The vast majority of genes (97.88%; 47,135 of 48,158) were anchored on chromosomes, and only 2.12% (1023 of 48,158) were located on scaffolds. We used the newly assembled NHCC001 genome as a reference to measure the expression levels of 48,159 annotated genes (Supplementary Table S18) and found that 25,056 and 21,662 genes were expressed in leaf and root tissues, respectively, with FPKM ≥ 1.

Homology-based and de novo approaches were also used to search for and predict repetitive sequences. A total of 213.04 Mb (52.56%) of the assembled NHCC001 genome comprised repetitive sequences, a percentage higher than that of the previous *B. rapa* Chiifu v3.0 assembly (37.93%, 133.95 Mb) (Supplementary Table S8). Among these repetitive elements, LTR retrotransposons were the most abundant, accounting for 20.42% of the genome, followed by DNA transposons (5.37%), LINEs (3.20%), and SINEs (0.29%) (Supplementary Table S8). In addition, 1.19% (4.86 Mb) of the assembled genome was annotated as non-coding RNA, including 151 miRNAs, 1,361 tRNAs, 3,907 rRNAs, and 1,272 snoRNAs.

### Evolution of the NHCC001 genome

**Fig. 2:**
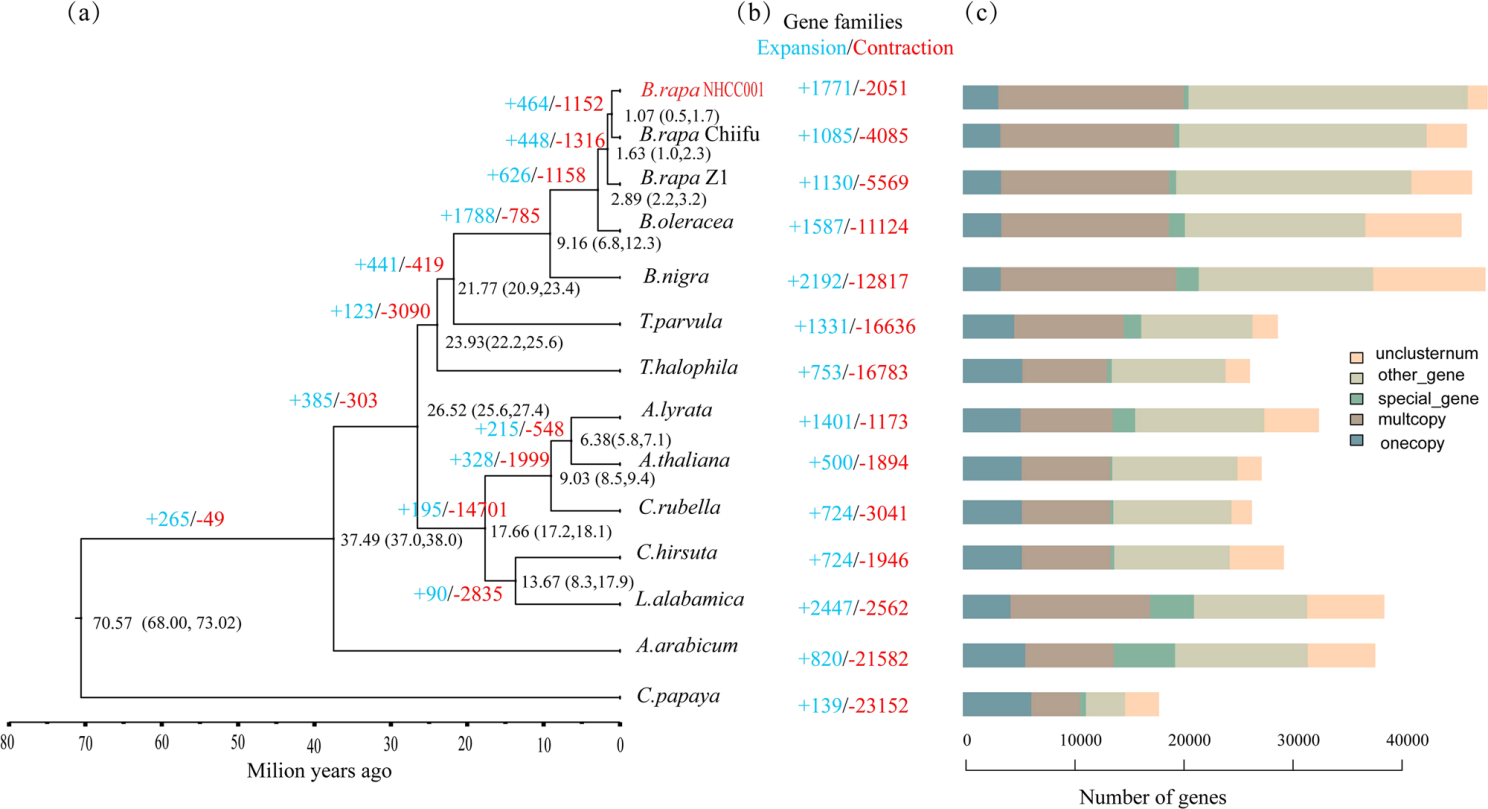
Phylogenetic tree and gene family changes in NHCC001 and related species. **a** A phylogenetic tree constructed from single-copy gene families in NHCC001 and 13 additional plants. The estimated divergence times (million years ago, Mya) are indicated at each node, representing 95% credibility intervals of the estimated dates. **b** Expansions and contractions of gene families. Gene family expansions and contractions are indicated by the numbers in blue and red, respectively. **c** Clusters of orthologous and paralogous gene families in NHCC001 and 13 additional species

The genome sequences of 11 plant species and two other *B. rapa* subspecies were collected and used for comparative genomic analysis with NHCC001 to investigate its genome evolution and divergence. We clustered the annotated NHCC001 genes with those from the other plant genome using OrthoMCL. Of the 48,158 protein-coding genes in the NHCC001 genome, 46,316 were grouped into 27,536 gene families with an average of 1.68 genes per family. There were 7835 common gene families and 165 NHCC001-specific families (Fig. 2c, Supplementary Table S9). Furthermore, we found that 1771 gene families had expanded and 2051 had contracted in the newly assembled NHCC001 genome (Fig. 2b). The 165 NHCC001-specific gene families contained 423 genes, and 1501 of the 7835 common gene families contained one copy in each plant. We used these 1501 single-copy orthologs for phylogenetic analysis based on the maximum likelihood method. *B. rapa* NHCC001 and *B. rapa* Chiifu, which derive from the common ancestral genome of *Brassica* species, were clustered together on a branch (Fig. 2a). Using *B. oleracea* as an outgroup, evolutionary rates and protein mutation sites were compared among the three *B. rapa* genomes to add confidence to the genomic analysis (Supplementary Fig. S4). The three subgenomes of *B. rapa* diverged approximately 1.0–2.3 Mya, and the data further confirmed that the *Brassica* ancestor diverged from *Thellungiella* *parvula* approximately 20.9–23.4 Mya (Fig. 2a).

**Fig. 3:**
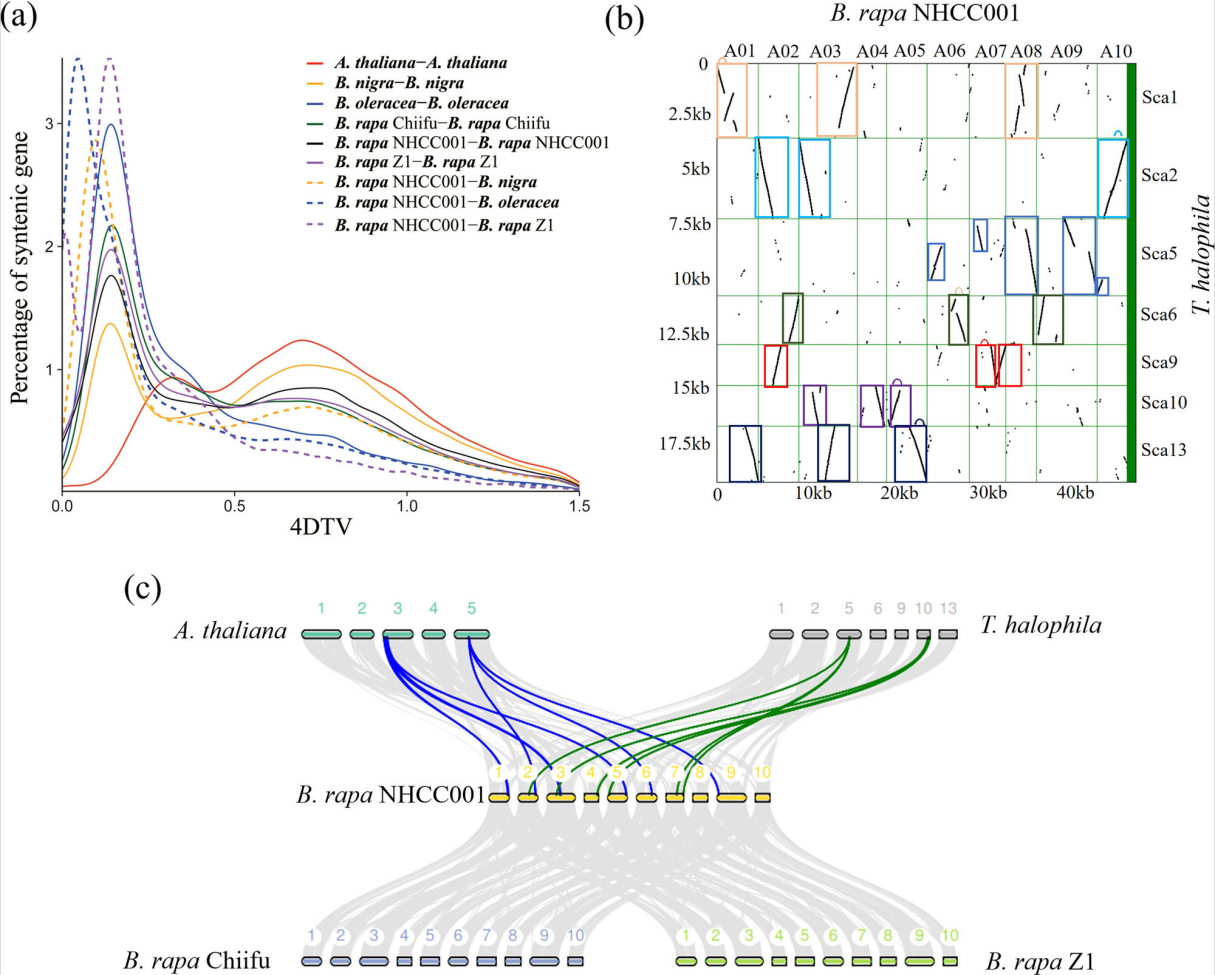
Evolution of the NHCC001 genome. **a** Four-fold synonymous third-codon transversion rate (4Dtv). 4dTv distance distribution of duplicated gene pairs in syntenic blocks within the genomes of *B. rapa* NHCC001, *B. rapa* Chiifu, *B. rapa* Z1, *B. oleracea*, *B. nigra*, and *A. thaliana*. **b** Segmental collinearity of the *B. rapa* NHCC001 and *T. halophila* genomes. Conserved collinear blocks of gene models between the ten chromosomes of *B. rapa* NHCC001 (horizontal axis) and the eight longest scaffolds of *T. halophila* (vertical axis) are shown. **c** Syntenic blocks between *B. rapa* NHCC001 and four other *Brassicaceae* species. The numbers indicate the corresponding chromosomes or scaffolds in each species

4DTv and Ks values of gene pairs were estimated based on the syntenic blocks detected among NHCC001 and other plant genomes (Fig. 3a, Supplementary Fig. S3). The results indicated that a recent WGT event at 4Dtv ≈ 0.15 (Ks peak value ≈ 0.3), which was previously reported as a *Brassicaceae*-specific triplication (Br-α-WGD)^13–15^, had also occurred in the evolutionary history of NHCC001 (Fig. 3a). Furthermore, we confirmed the almost complete triplication of the *B. rapa* NHCC001 genome relative to those of *Thellungiella* *halophila* and *A. thaliana* (Fig. 3b–c). The divergence of *B. rapa* NHCC001 and *B. nigra* occurred at a peak of ∼0.10, followed by that of *B. rapa* NHCC001 and *B. oleracea* (4dTv = 0.05), consistent with the phylogenetic analysis (Fig. 3). The genomes of *B. rapa* NHCC001, *B. rapa* Z1, and *B. rapa* Chiifu arose recently and did not exhibit significant divergence compared with the A and B genomes of *Brassica* species (Supplementary Fig. S3). These findings were consistent with the phylogenetic analysis of *Brassica* and other representative species.

### Chromosome structure of the *B. rapa* NHCC001 genome

**Fig. 4:**
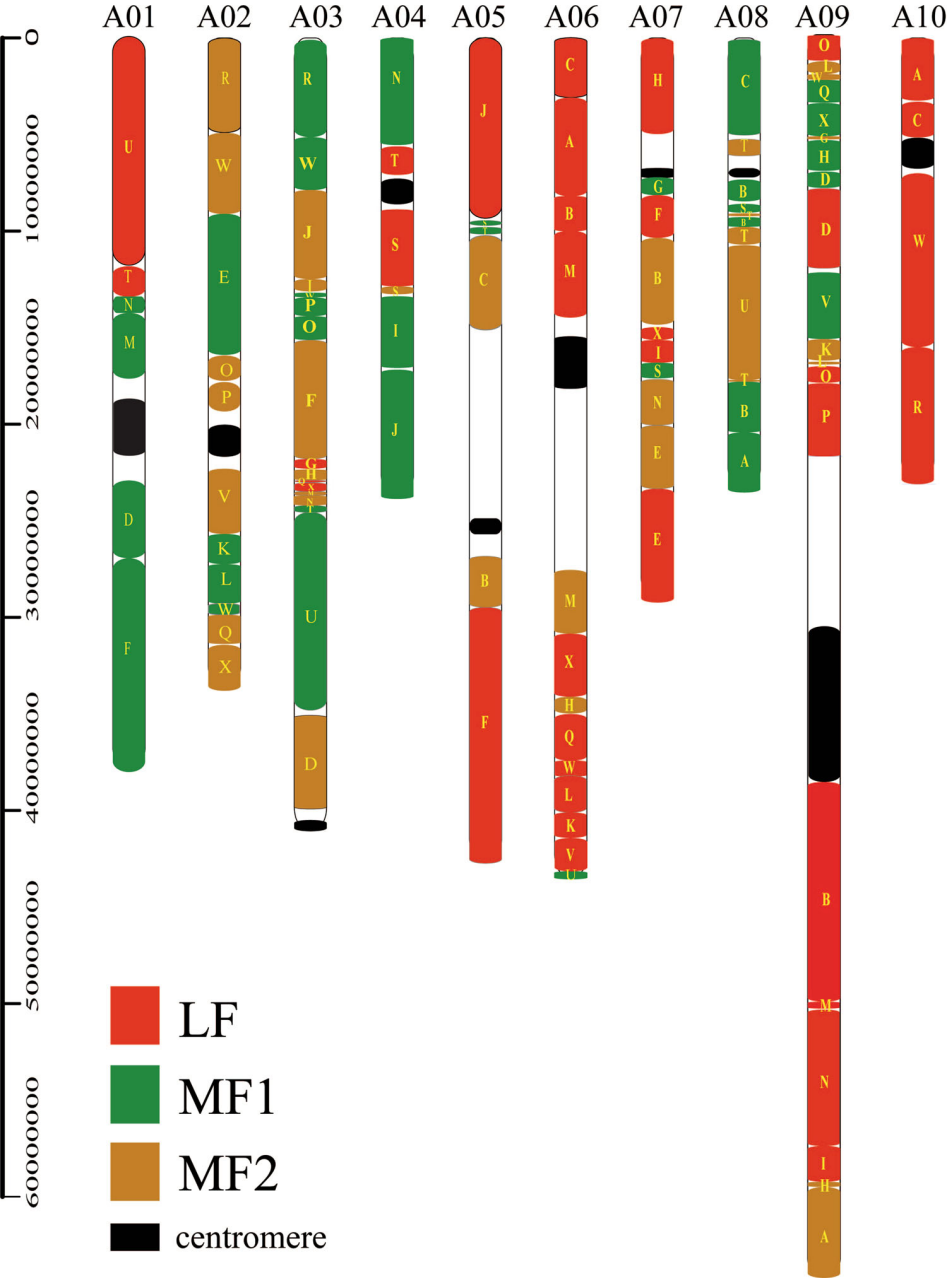
Distribution of genomic blocks along the ten chromosomes of the *B. rapa* NHCC001 genome. Genome blocks were assigned to the subgenomes LF, MF1, and MF2. Centromeres are shown as black ovals

Previous studies have proposed a model for comparative genomics and chromosomal analyses based on the concept of the Ancestral Crucifer Karyotype (ACK; *n* = 8), which comprises eight chromosomes and 24 genomic blocks (GBs, named A to X)^16^. Syntenic orthologs between NHCC001 and *A. thaliana* were identified first^17^, and the three subgenomes were identified based on the syntenic relationship between NHCC001 and *A. thaliana* (Supplementary Table S19). Subgenomes were ordered based on gene densities from high to low and were named LF, MF1, and MF2^17^. As expected, all 72 genome blocks (3 × 24) in the NHCC001 genome were identified, compared with 71 identified in Chiifu v3.0 and previous versions^18^ (Fig. 4). Compared with the distribution of genome blocks in Chiifu V3.0, most were arranged similarly in NHCC001. The lost genome block G (MF2) in Chiifu was identified on chromosome A09 between X(MF1) and H(MF1) in NHCC001 (Fig. 4, Supplementary Table S20). In addition, we identified two new fragmented genome blocks: T(MF1) and O(LF) were identified on chromosomes A05 and A09, respectively, but were not observed in Chiifu V3.0. Two new fragmented genome blocks, F (LF) and F (MF1) on chromosomes A01 and A05 in Chiifu V3.0, were not identified in NHCC001 or in ref. ^10,18^. We counted the gene numbers in the three subgenomes and found 13,283, 9011, and 7419 genes in the LF, MF1, and MF2 subgenomes, respectively, that were syntenic to *A. thaliana*.

### Global genome comparisons of three *B. rapa* genomes

SNPs and indels were identified between *B. rapa* NHCC001, *B. rapa* Z1, and *B. rapa* Chiifu (Fig. 1, Supplementary Table S10). A total of 1,718,037 SNPs and 738,275 indels were identified between *B. rapa* NHCC001 and *B. rapa* Z1, and 1,305,874 SNPs and 469,629 indels were identified between *B. rapa* NHCC001 and *B. rapa* Chiifu. We then compared the NHCC001 genome sequence to those of Chiifu and Z1 and identified a large number of syntenic regions (Fig. 3c). A total of 534 syntenic blocks were detected between *B. rapa* NHCC001 and *B. rapa* Chiifu, comprising 49,166 gene pairs. Likewise, 557 syntenic blocks were detected between *B. rapa* NHCC001 and *B. rapa* Z1, comprising 48,976 gene pairs (Fig. 3c). Tandem gene arrays were also identified in the three genomes using SynOrths^19^. A total of 2211 tandem arrays (corresponding to 5296 tandemly duplicated genes) were identified in *B. rapa* NHCC001. By contrast, more tandem arrays (2317 arrays, 5584 genes) were identified in the *B. rapa* Chiifu genome and fewer (2013 arrays, 4781 genes) in the Z1 genome.

We identified 10,851 NHCC001-specific genomic segments (~13 Mb) and 8496 Chiifu-specific genomic segments (~10 Mb) longer than 500 bp (Supplementary Table S11). Most (98.7%) of these PAV (presence and absence variation) sequences were shorter than 5 kb, although 222 and 177 PAV sequences were longer than 5 kb in NHCC001 and Chiifu, respectively (Supplementary Table S11). Three NHCC001-specific sequence clusters (compared with Chiifu) on chromosomes 7 and 2 contained 132 predicted genes, and eight NHCC001-specific sequence clusters (compared with Z1) on eight separate chromosomes contained 596 predicted genes (Supplementary Table S12). Details of PAV sequences and clusters identified between *B. rapa* NHCC001 and *B. rapa* Z1 are shown in Supplementary Table S13. Among the PAV genes identified between NHCC001 and Chiifu, 125 were specific to NHCC001, and 369 were specific to Chiifu (Supplementary Table S21). These specific segments and genes may contribute to the diversity of the three *B. rapa* subspecies.

### Leaf adaxial-abaxial patterning genes in *B. rapa*

The most significant difference between NHCC001, a non-heading Chinese cabbage cultivar, and Chiifu, the heading Chinese cabbage, is the heading trait. Leaf incurvature, controlled by multiple genes, is an essential prerequisite for the formation of a leafy head^20,21^. Previous studies have identified adaxial-abaxial (ad-ab) patterning genes and investigated their genetic variation to uncover the mechanisms that underlie leaf incurvature during head formation in heading *B. rapa* and *A. thaliana*^22^. Using 26 homologs from *A. thaliana*, we identified 51, 47, and 49 leaf ad-ab patterning genes in NHCC001, Chiifu v3.0, and Z1, respectively (Supplementary Table S14). Copy number variation was found among the three *B. rapa* genomes. In non-heading NHCC001 and Z1, three homologs of *AtARF4* were identified (Supplementary Table S14), compared with only two *AtARF4* homologs in heading Chinese cabbage Chiifu. Previous studies have shown that *arf3 arf4* double mutants develop leaves that are curled up and resemble the phenotype of *kan1 kan2* leaves, indicating an overlap in the function of leaf abaxial polarity^23–25^. Furthermore, there were two homologs of *AtAGO7* in NHCC001 and Z1 but only one *AtAGO7* homolog in Chiifu. Two *AtDCL1* homologs in Chiifu were identified as tandem duplicates. Copy number variation in ab-ad genes may therefore contribute to leaf head formation in Chinese cabbage.

### Identification of genes involved in ascorbic acid and glucosinolate metabolic pathways

**Fig. 5:**
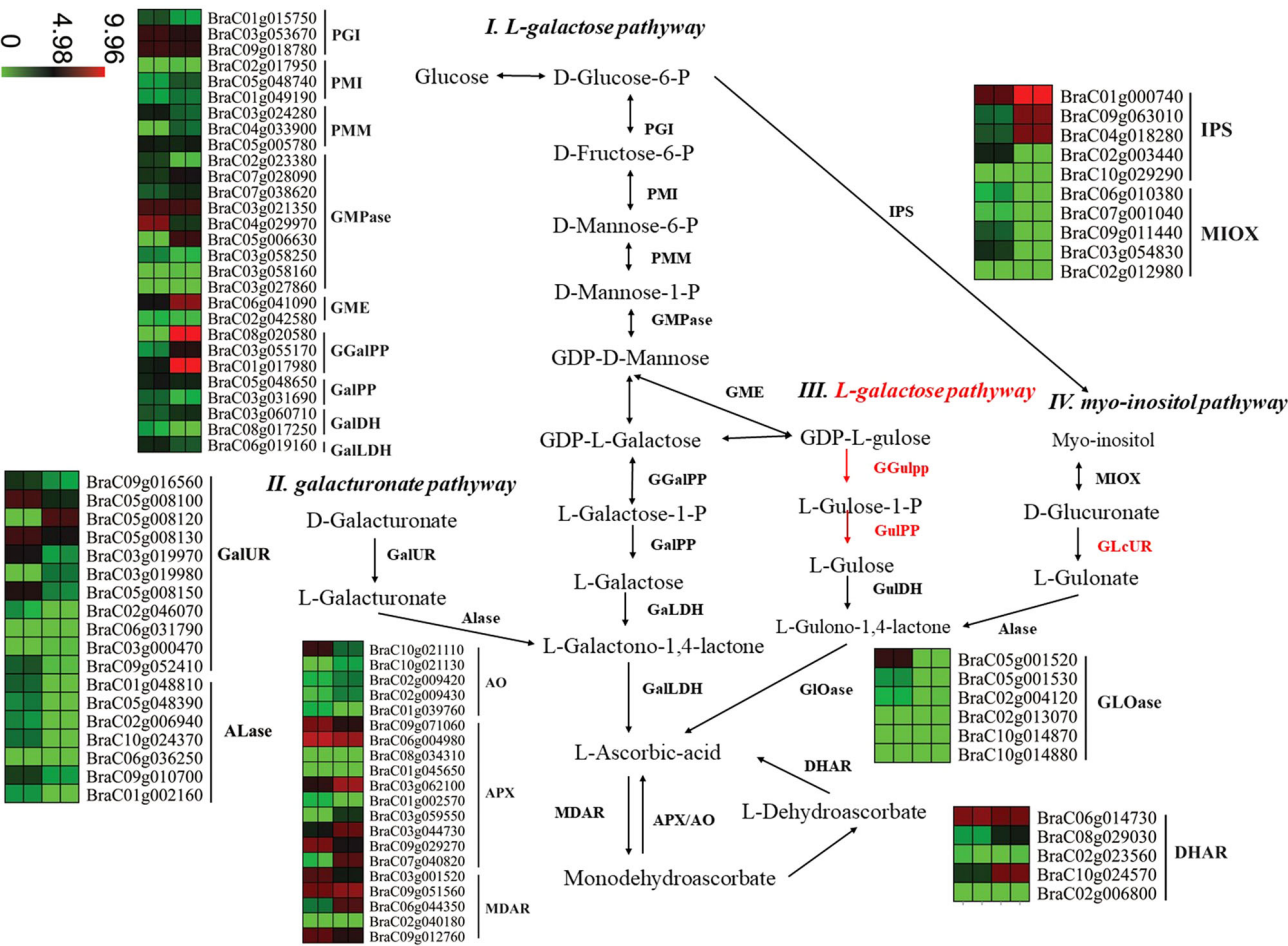
Genes involved in the biosynthesis and recycling of ascorbic acid (AsA, vitamin C) and their expression patterns in *B. rapa* NHCC001. Red lines indicate hypothetical reactions. Heatmaps show log2-transformed expression values of ASA-related genes in Root-1, Root-2, Leaf-1, and Leaf-2 (from left to right)

Pak-choi is well known for its high nutritional value, particularly its abundant contents of ascorbic acid (AsA, vitamin C) and glucosinolates (GSLs). A previous study reported that the leaf AsA concentration in NHCC001 was ~110 mg/100 g FW^26^. Using the newly assembled NHCC001 genome and the sequences of AsA-related genes from *A. thaliana*, we identified and compared genes involved in AsA biosynthesis and recycling from *B. rapa* NHCC001, *B. rapa* Chiifu, and *B. rapa* Z1 (Fig. 5, Supplementary Table S15). A total of 87, 93, and 93 AsA-related genes were identified in the *B. rapa* NHCC001, Chiifu, and Z1 genomes, respectively (Supplementary Table S15). Four regions of homeologs had undergone tandem duplication. The numbers of AsA-related homologs were highly consistent among the three *B. rapa* genomes; most were located on conserved collinear blocks and showed little variation among the three subspecies. The expression patterns of these genes were measured in the root and leaf tissues of NHCC001 (Fig. 5). Two *GGalPP* homologs and one *IPS* homolog were highly expressed in the leaf (Fig. 5), whereas some *APX*, *MDAR*, and *DHAR* homologs from the recycling pathway were highly expressed in both roots and leaves (Fig. 5).

**Fig. 6:**
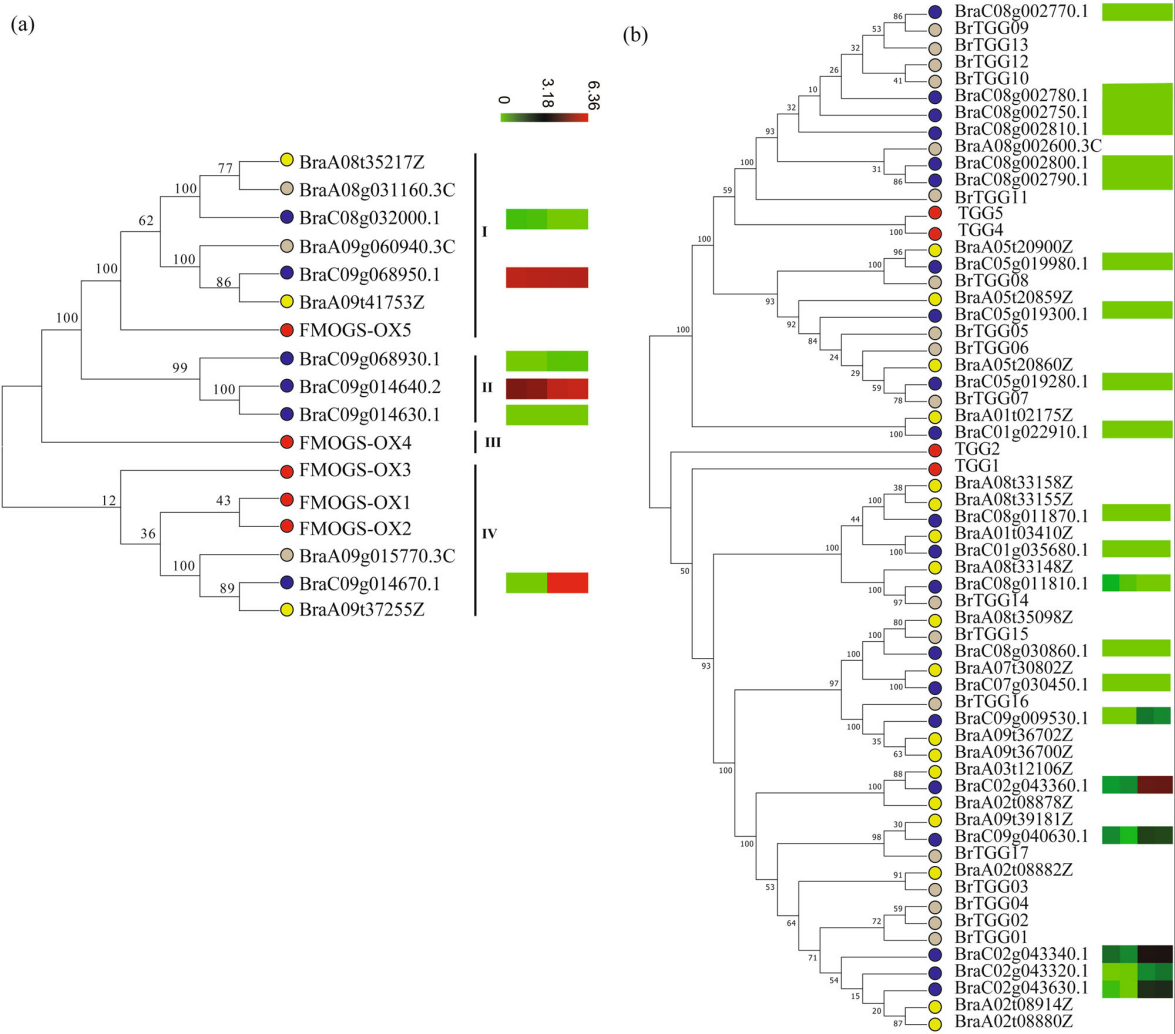
Phylogenetic relationships and expression patterns of the *FMOGS-OX* and *TGG* genes. Phylogenetic tree of the *FMOGS-OX* (**a**) and *TGG* (**b**) genes in *B. rapa* NHCC001 and three related species, and their expression patterns in *B. rapa* NHCC001. The NJ tree was constructed with MEGA using the full-length amino acid sequences. The solid red, blue, gray, and yellow dots represent *A. thaliana*, *B. rapa* NHCC001, *B. rapa* Chiifu, and *B. rapa* Z1, respectively. Heatmaps show log2-transformed expression values of FMOGS-OX and TGG genes in Root-1, Root-2, Leaf-1, and Leaf-2 (from left to right)

Glucosinolates and their hydrolysis products have an important role in human health and plant defence^27^. More than 130 structurally distinct GSLs are present in 16 families of dicotyledonous angiosperms^27,28^, and more than 14 different GSLs have been identified and quantified in the young leaves of different *B. rapa* varieties^29^. Using *A. thaliana* GSL genes as queries, GSL genes were identified in *B. rapa* NHCC001, Chiifu, and Z1 (Supplementary Table S16). Interestingly, GSL gene numbers were highly expanded in the *B. rapa* genome, with a high proportion of tandem genes. GSL biosynthesis and catabolism are probably similar among the three sequenced *B. rapa* species, but there was nonetheless substantial variation in the proportion of tandem genes such as *MAM1*, *ST5b*, *FMOGS-OX*, *AOP*, *TGGs*, and *NSPs* (Supplementary Table S16). Flavin-monooxygenase FMOGS-OX catalyzes the S-oxygenation of methylthioalkyl to methylsulfinylalkyl GSLs during the biosynthesis of aliphatic GSLs in *Arabidopsis thaliana*^30^. We identified six, three, and three FMOGS-OX homologs in NHCC001, Chiifu, and Z1, respectively (Supplementary Table S16). Phylogenetic analysis showed that the FMOGS-OX genes were clustered into four clades, one of which included only three FMOGS-OX genes from *B. rapa* NHCC001 (Fig. 6a). Gene expression analysis showed that *BraC09g068950* in clade I and *BraC09g014640* in clade II were highly expressed in both roots and leaves, whereas *BraC09g014670* had the highest leaf expression of all the *FMOGS-OX* genes. TGG catalyzes the hydrolysis of GSLs into compounds that are toxic to various microbes and herbivores^31^. TGG homologs were identified in the three *B. rapa* genomes and used to construct a phylogenetic tree (Fig. 6b, Supplementary Table S16). To our surprise, only one TGG homologue was initially identified in the *B. rapa* Chiifu v3.0 genome, despite the fact that nine TGG homologs were identified in the previous *B. rapa* Chiifu genome version^5^. We speculated that this may have been caused by a gene prediction error in Chiifu v3.0. We therefore searched the Chiifu v3.0 genome using four *A. thaliana* TGGs as queries and found 17 new TGG genes. Detailed information on these genes is provided in Supplementary Table S17. There were 21 TGGs in *B. rapa* NHCC001. Two tandem duplicates, *BraC02g043340* and *BraC02g043360*, both showed high expression in NHCC001 leaves (Fig. 6b).

## Discussion

*Brassica rapa* species can be grouped into six subspecies: turnips, sarsons, turnip rapes, and the Japanese group, which includes pak-choi and heading Chinese cabbages^32^. Although they originated from the same ancestor, these varieties show very high morphotype diversity. To date, only one cultivar of heading Chinese cabbage (Chiifu-401-42)^10^ and one sarson type (Z1)^12^ have been sequenced. The scarcity of genomic resources has hindered research on the evolution of non-model plant species and the genetic basis of phenotypic diversity. In the present study, we assembled a high-quality genome of the pak-choi cultivar ‘Suzhouqing’ (NHCC001) using a combination of PacBio and Hi-C data. The assembly covers approximately 84.84% of the estimated NHCC001 genome. Use of PacBio and Hi-C technologies provided a high-quality assembly in terms of contiguity and completeness of genic and repetitive regions. More repeat sequences (213.04 Mb) were identified in the present study than in previously published *B. rapa* genomes (Table 1). A total of 48,158 genes were identified, more than those identified in the *B. rapa* Chiifu (45,985)^11^ and *B. rapa* Z1 (46,721)^12^ assemblies. Our assembly represents a real improvement of the *B. rapa* genome, particularly for regions enriched in repetitive elements, and provides a valuable resource for comparative genomics and evolutionary studies.

Many efforts have been made to resolve the relationships among subspecies and the domestication history of *B. rapa*. Chen et al. and Qi et al. also suggested that the Chinese cabbage group was positioned at the most distant point from the *B. rapa* root and was most divergent from the last *B. rapa* common ancestor^2,32^. Our phylogenetic analysis further confirmed that the sarson type diverged from the last *B. rapa* common ancestor earlier than Chinese cabbage and pak-choi (Fig. 2). Comparative genomic analysis revealed variations in the three *B. rapa* genomes; numerous intraspecific variations such as SNPs, indels, and PAVs were revealed. Copy number variation was also identified in adaxial-abaxial patterning genes, which may lead to a better understanding of the molecular mechanisms that underlie the leaf heading trait in *B. rapa*. Based on homolog searches, we identified candidate AsA-related and GSL-related genes in the three *B. rapa* genomes. This more complete genome assembly will provide a solid basis for future metabolism-related studies.

## Conclusion

Our newly assembled *B. rapa* genome achieves a high level of continuity and genic completeness. The new *B. rapa* genome will be of great help in understanding the evolution of the *Brassicas* and will provide an important resource for research, especially the molecular investigation of agricultural traits and the breeding of *B. rapa* ssp. *chinensis*.

## Materials and methods

### Illumina sequencing of short paired-end (PE) reads

The *Brassica campestris* (syn*. Brassica rapa*) ssp*. chinensis* (‘Suzhouqing’, green petiole type pak-choi) self-inbred line NHCC001 was used for genome sequencing. Genomic DNA was extracted from NHCC001 leaf tissue and fragmented. One library was constructed and sequenced on the Illumina platform (Illumina, San Diego, CA, USA), and 76.35 Gb of clean reads were generated after trimming adaptors and low-quality bases and removing mitochondria and chloroplast DNA contamination. These data were used for genome estimation, gap closing, assembly polishing, and completeness assessment of the final assembly. Genome size was estimated using the formula: Genome Size=total *k*-mer number/average peak depth using *k*-mer analysis.

### RNA sequencing

Root, stem, leaf, and flower tissues were harvested and immediately frozen in liquid nitrogen. Total RNA was extracted using the TRIzol reagent (Invitrogen, USA) following the manufacturer’s instructions and pooled for sequencing. SMRTbell libraries were constructed using the PacBio DNA Template Prep Kit 2.0 and then sequenced on the PacBio RS II platform. In addition, cDNA libraries were constructed from roots and leaves and used for second-generation sequencing on the Illumina HiSeq 2500 platform to generate 125-bp paired-end reads following the manufacturer’s protocol.

### Pacific Biosciences (PacBio) sequencing and de novo assembly of PacBio subreads

Genomic DNA was isolated and extracted from fresh NHCC001 leaves using the CTAB method^33^. Quality control of genomic DNA was performed using a Nanodrop spectrophotometer. 10 ug of gDNA was sheared to ~20-kb targeted size using a Covaris g-TUBE, and a 20-kb insert library was constructed following the standard PacBio protocol. SMRT cells were sequenced on the PacBio RSII platform (PacBio Sequel sequencer at Biomarker Technologies Corporation) with P6-C4 chemistry. PacBio subreads were corrected, trimmed, and assembled using SMARTdenovo (5cc1356) (https://github.com/ruanjue/smartdenovo.git). Sequencing errors in subreads were corrected using Canu (v1.5)^34^ with the settings genomeSize = 480,000,000, corOutCoverage = 100, and correctedErrorRate = 0.025. Finally, to ensure base-pairing accuracy of the assembly results, we further polished the consensus sequence based on error-corrected PacBio subreads using Arrow (v2.3.3) with the parameter -j 4 and Illumina PE reads using Pilon (v1.22)^35^ with the parameters --mindepth 10, --changes, --threads 4, and --fix bases.

### Hi-C library sequencing

Hi-C libraries were prepared from NHCC001 leaves as described previously^36^ and sequenced on the Illumina HiSeq X Ten platform (2×150 bp) to generate 229,427,069 paired-end reads. The Hi-C data were mapped to PacBio-based contigs using BWA (v0.7.10-r789; aln mapping method) with the parameters -M 3, -O 11, -E 4, and -t 8. HiC-Pro (v2.8.1)^37^ was used for duplicate removal and quality control. Using contigs assembled from PacBio data, Hi-C data were used to correct mis-joins in contigs and to order and orient contigs. Pre-assembly was performed for contig correction by splitting contigs into segments and then pre-assembling the segments with Hi-C data. Misassembled points were defined and broken when split segments could not be placed into the original position. Finally, the corrected contigs were assembled using LACHESIS with parameters CLUSTER_MIN_RE_SITES = 30, CLUSTER_MAX_LINK_DENSITY = 2, ORDER_MIN_N_RES_IN_TRUN = 25, and ORDER_MIN_N_RES_IN_SHREDS = 26 with Hi-C valid pairs. Gaps between ordered contigs were filled with 100 Ns. To improve the contiguity of the assembly results, a gap-closing step for pseudochromosomes was performed using PBJelly (v15.2.20)^38^ with the parameter --minGap=1 using error-corrected Nanopore clean reads. The contact map was visualized with heatmaps at a 100-kb resolution.

### Genome assembly evaluation

The completeness of the assembly was evaluated based on the full-length transcriptome and PE reads using Tophat2^39^ with default parameters and BWA (v0.7.10)^40^ with parameters -t 4 and -M, respectively. The corrected PacBio subreads were also used for genome evaluation with blasr (v1.3.1) (https://github.com/PacificBiosciences/blasr) using parameters -bestn 1, -minPctIdentity 70, and -nproc 4. BUSCO v4.0.6 and CEGMA (v2.5) were also used to assess assembly completeness. BUSCO^41^ was run using the embryophyta_odb10 dataset with default parameters.

### Annotation of transposable elements (TEs)

Transposable elements were identified using de novo and homology-based methods. A de novo repetitive element database was built using four de novo software packages, including RepeatScout (v1.0.5)^42^, LTR_FINDER (v1.05)^43^, MITE-Hunter (20100819)^44^, and PILER-DF (v1.0)^45^. The de novo library was merged with Repbase 19.60^46^ and classified into different categories with the PASTEClassifier.py^47^ script embedded in REPET (v2.5)^48^. RepeatMasker (version open-4.0.5)^49^ was used to identify repetitive elements using the combined library with parameters -nolow, -no_is, -norna, -engine wublast, -qq, and -frag 20000.

### Gene prediction and functional annotation

GeMoMa (v1.3.1)^50^ was used for homology prediction with the parameter evalue = 0.00001. Five de novo gene prediction software packages were used, including Genscan (hollywood.mit.edu/GENSCAN.html), Augustus (v2.4)^51^, GeneID (1.4)^52^, SNAP (v2006-07-28)^53^ and GlimmerHMM (v3.0.4)^54^. Parameters in Augustus were trained with unigenes assembled from pooled RNA-seq data. For RNA-seq based prediction, NGS transcripts and full-length transcripts were used. NGS transcripts were assembled using HISAT (v2.0.4)^55^ and StringTie v1.2.3)^56^ and then aligned to the genome assembly using BLAT^57^ with the parameters identity ≥ 0.95 and coverage ≥ 0.90. Unigenes were filtered using PASA (v2.0.4)^58^. Clean RNA-seq reads were mapped to the assembled genome using TopHat^39^, and transcripts were assembled using Cufflinks (v2.1.1)^39^. TransDecoder (v2.0)^59^ and GeneMarkS-T (v5.1)^60^ were used to identify the gene structure. EVidenceModeler (EVM, v1.1.1)^61^ was used to obtain an integrated gene set from the three prediction strategies above with different weight settings. The final gene set was obtained after filtering out coding sequences (CDS) shorter than 300 bp with frameshift mutations or premature stop codons. Functional annotation of the final gene set was performed using BLASTP (E-value 1e^−5^) embedded in the blast+ package (v2.2.31)^62^ against multiple databases, including KEGG^63^, Swiss-Prot^64^, TrEMBL^64^, and NCBI nr^65^. GO annotations were assigned using the BLAST2GO pipeline (v2.5)^66^. The newly annotated genes were named based on the following conventions: Bra for *Brassica rapa*, followed by C for *chinensis*, then the chromosome number and the letter “g” for gene. The six digits after “g” were assigned based on the gene’s position relative to the top of the chromosome.

### Non-coding RNA (ncRNA) predictions

tRNAscan-SE (v2.0)^67^ was used to predict tRNAs with two embedded searching methods (tRNA-scan and EufindtRNA). tRNAs located in repetitive regions were excluded, and tRNAs with prediction scores over 20 were retained. miRNAs were identified by a homology search against miRBase (release 22)^68^ with one mismatch allowed. miRDeep2^69^ was used to predict secondary structures, and miRNAs with hairpin structure were retained. Other ncRNAs were predicted based on an Infernal (v1.1.2)^70^ search against the Rfam (v12.1)^71^ database with default parameters.

### Pseudogene prediction

GenBlastA (v1.0.4)^72^ with the parameter -e 1e-5 was used to identify homologous sequences in the genome, and Genewise (v2.4.1)^73^ with the parameters -both and -pseudo was used to identified pseudogenes when premature stop codons or frameshift mutations were present in homologous sequences with 60% identity and 60% coverage.

### Gene family clustering

Proteins from 14 genomes were used for gene family clustering. Proteins from *A. thaliana* (TRAI10.1) were downloaded from https://www.arabidopsis.org/. Proteins from *Brassica nigra*, *Thellungiella parvula*, *Thellungiella halophila*, *Leavenworthia alabamica*, *Capsella rubella*, *Brassica oleracea* (v1.1), *Brassica rapa* Chiifu (v3.0), and *Aethionema arabicum* were downloaded from http://brassicadb.org/brad/datasets/pub. Proteins from *Cardamine hirsuta* (v1.0) were downloaded from http://chi.mpipz.mpg.de/assembly.html. Proteins from *Brassica rapa* Z1 were downloaded from http://www.genoscope.cns.fr/plants. Proteins from *Carica papaya* (Papaya1.0) and *Arabidopsis lyrata* subsp. *lyrata* (v.1.0) were downloaded from NCBI. Only the longest transcript of each protein was used. OrthoMCL (v2.0.9; mcl inflation factor 1.5)^74^ was used to cluster gene families. All-against-all BLASTP searches (Blast + version 2.3.0)^62^ were performed with a *P*-value cutoff of 1e^−5^ and a minimum match length of 50%.

### Phylogenetic tree construction and divergence time estimation

Single-copy genes were aligned using MUSCLE (v3.8.31)^75^ and concatenated into one super-gene sequence for each plant genome. A maximum likelihood phylogenetic tree was constructed from the aligned protein sequences using PhyML 4.0^76^ with the parameters --sequential, --multiple 1, --pars, --bootstrap 100, --model JTT, -f m, -t e, --pinv e, --nclasses 4, --use median, and --no_memory_check. MCMCTree implemented in the PAML package (v4.7b)^77^ was used to estimate speciation times.

### Expansion and contraction of gene families

CAFE (v2.0)^78^ was used to infer gene family sizes in the most recent common ancestor (MRCA) and to determine the significance of gene family expansion/contraction based on the phylogenetic tree topology. The birth and death parameter (λ) was 0.002, and the *P*-value was 0.01.

### Synteny and 4DTv analysis

The BLASTP program was used to identify orthologous and paralogous genes. MCscanX^79^ was used to recognize syntenic blocks with parameters E_VALUE = 1e−05, MAX GAPS = 25, and MATCH_SIZE = 5. Syntenic blocks were visualized with MCscan, and chromosome lengths were not scaled. The 4DTv value of each gene pair was calculated and then corrected using the HKY model^80^. The Ks value of each syntenic gene pair was calculated using the yn00 program in the PAML package^77^. Chromosome-scale syntenic block plots and dotplot were constructed using the python version of MC scan (https://github.com/tanghaibao/jcvi/wiki/MCscan).

### Identification of PAV sequences and PAV clusters

PAV sequences in the genomes of *B. rapa* NHCC001, *B. rapa* Chiifu, and *B. rapa* Z1 were identified using a sliding-window method as described previously^81^.

### Identification of ascorbic acid-related and glucosinolate-related genes

*A. thaliana* AsA-related and GSL-related genes have been reported and were used as the set of reference genes in this study^5,82–86^. Their protein sequences were aligned with corresponding protein sets from the *B. rapa* genome using BLASTP (E-value ≤ 1 × 10^−10^, identity ≥55).

## Supplementary information

Supplementary Table S1-S17

Supplementary Table S18

Supplementary Table S19

Supplementary Table S20

Supplementary Table S21

Supplementary Figure S1

Supplementary Figure S2

Supplementary Figure S3

Supplementary Figure S4

Supplementry information

## Data Availability

All of the Illumina and Nanopore sequencing data have been deposited in the Sequence Read Archive database under NCBI BioProject ID PRJNA645752. The whole-genome assembly and annotation data are publically available at: https://www.tbirs.cn/NHCCDB/Genome.jsp.

## References

[1] Nagahara U (1935). Genome analysis in *Brassica* with special reference to the experimental formation of *B. napus* and peculiar mode of fertilization. Jpn. J. Bot..

[2] Qi X (2017). Genomic inferences of domestication events are corroborated by written records in *Brassica rapa*. Mol. Ecol..

[3] Karam MA, Morsi YS, Sammour RH, Ali MR (2010). Assessment of genetic relationships within *Brassica rapa* subspecies based on polymorphism. Int. J. Curr. Microbiol. Appl. Sci..

[4] Arabidopsis Genome Initiative. (2000). Analysis of the genome sequence of the flowering plant *Arabidopsis thaliana*. Nature.

[5] Yu J (2002). A draft sequence of the rice genome (*Oryza sativa* L. ssp. *indica*). Science.

[6] Chen F (2018). The sequenced angiosperm genomes and genome databases. Front Plant Sci..

[7] Treffer R, Deckert V (2010). Recent advances in single-molecule sequencing. Curr. Opin. Biotechnol..

[8] Jiao Y (2017). Improved maize reference genome with single-molecule technologies. Nature.

[9] Schmidt MH (2017). De novo assembly of a new *Solanum pennellii* accession using nanopore sequencing. Plant Cell.

[10] Wang X (2011). The genome of the mesopolyploid crop species *Brassica rapa*. Nat. Genet..

[11] Zhang L (2018). Improved *Brassica rapa* reference genome by single-molecule sequencing and chromosome conformation capture technologies. Hortic. Res..

[12] Belser C (2018). Chromosome-scale assemblies of plant genomes using nanopore long reads and optical maps. Nat. Plants.

[13] Franzke A, Lysak MA, Al-Shehbaz IA, Koch MA, Mummenhoff K (2011). Cabbage family affairs: the evolutionary history of Brassicaceae. Trends Plant Sci..

[14] Lagercrantz U, Lydiate DJ (1996). Comparative genome mapping in *Brassica*. Genetics.

[15] Tank DC (2015). Nested radiations and the pulse of angiosperm diversification: increased diversification rates often follow whole genome duplications. New Phytol..

[16] Schranz ME, Lysak MA, Mitchell-Olds T (2006). The ABC’s of comparative genomics in the *Brassicaceae*: building blocks of crucifer genomes. Trends Plant Sci..

[17] Cheng F (2012). Biased gene fractionation and dominant gene expression among the subgenomes of *Brassica rapa*. PloS ONE.

[18] Cheng F (2013). Deciphering the diploid ancestral genome of the Mesohexaploid *Brassica rapa*. Plant Cell.

[19] Cheng F, Wu J, Fang L, Wang X (2012). Syntenic gene analysis between *Brassica rapa* and other *Brassicaceae* species. Front Plant Sci..

[20] Yu X (2013). QTL mapping of leafy heads by genome resequencing in the RIL population of *Brassica rapa*. PloS ONE.

[21] Mao Y (2014). MicroRNA319a-targeted *Brassica rapa* ssp. *pekinensis* TCP genes modulate head shape in Chinese cabbage by differential cell division arrest in leaf regions. Plant Physiol..

[22] Liang J, Liu B, Wu J, Cheng F, Wang X (2016). Genetic variation and divergence of genes involved in leaf adaxial-abaxial polarity establishment in *Brassica rapa*. Front Plant Sci..

[23] Pekker I, Alvarez JP, Eshed Y (2005). Auxin response factors mediate *Arabidopsis* organ asymmetry via modulation of KANADI activity. Plant cell.

[24] Eshed Y, Baum SF, Perea JV, Bowman JL (2001). Establishment of polarity in lateral organs of plants. Curr. Biol..

[25] Eshed Y, Izhaki A, Baum SF, Floyd SK, Bowman JL (2004). Asymmetric leaf development and blade expansion in *Arabidopsis* are mediated by KANADI and YABBY activities. Development.

[26] Ren J (2013). Comparison of ascorbic acid biosynthesis in different tissues of three non-heading Chinese cabbage cultivars. Plant Physiol. Biochem.

[27] Fahey JW, Zalcmann AT, Talalay P (2001). The chemical diversity and distribution of glucosinolates and isothiocyanates among plants. Phytochemistry.

[28] BrianClarke D (2010). Glucosinolates, structures and analysis in food. Anal. Methods.

[29] Yang B, Quiros CF (2010). Survey of glucosinolate variation in leaves of *Brassica rapa* crops. Genet Resour. Crop Evol..

[30] Li J, Hansen BG, Ober JA, Kliebenstein DJ, Halkier BA (2008). Subclade of flavin-monooxygenases involved in aliphatic glucosinolate biosynthesis. Plant Physiol..

[31] Rask L (2000). Myrosinase: gene family evolution and herbivore defense in *Brassicaceae*. Plant Mol. Biol..

[32] Cheng F (2016). Subgenome parallel selection is associated with morphotype diversification and convergent crop domestication in *Brassica rapa* and *Brassica oleracea*. Nat. Genet..

[33] Murray MG, Thompson WF (1980). Rapid isolation of high molecular weight plant DNA. Nucleic Acids Res..

[34] Koren S (2017). Canu: scalable and accurate long-read assembly via adaptive k-mer weighting and repeat separation. Genome Res..

[35] Walker BJ (2014). Pilon: an integrated tool for comprehensive microbial variant detection and genome assembly improvement. PLoS ONE.

[36] Xie T (2015). De Novo Plant genome assembly based on chromatin interactions: a case study of *Arabidopsis thaliana*. Mol. Plant.

[37] Servant N (2015). HiC-Pro: an optimized and flexible pipeline for Hi-C data processing. Genome Biol..

[38] English AC (2012). Mind the gap: upgrading genomes with Pacific Biosciences RS long-read sequencing technology. PLoS ONE.

[39] Trapnell C (2012). Differential gene and transcript expression analysis of RNA-seq experiments with TopHat and Cufflinks. Nat. Protoc..

[40] Li, H. Aligning sequence reads, clone sequences and assembly contigs with BWA-MEM. *Genomics* 1303. Preprint at https://arxiv.org/abs/1303.3997 (2013).

[41] Simão FA (2015). BUSCO: assessing genome assembly and annotation completeness with single-copy orthologs. Bioinformatics.

[42] Price AL, Jones NC, Pevzner PA (2005). De novo identification of repeat families in large genomes. Bioinformatics.

[43] Xu Z, Wang H (2007). LTR_FINDER: an efficient tool for the prediction of full-length LTR retrotransposons. Nucleic Acids Res..

[44] Han Y, Wessler SR (2010). MITE-Hunter: a program for discovering miniature inverted-repeat transposable elements from genomic sequences. Nucleic Acids Res..

[45] Edgar RC, Myers EW (2005). PILER: identification and classification of genomic repeats. Bioinformatics.

[46] Bao W, Kojima KK, Kohany O (2015). Repbase Update, a database of repetitive elements in eukaryotic genomes. Mob. DNA.

[47] Hoede C (2014). PASTEC: an automatic transposable element classification tool. PLoS ONE.

[48] Timothée F, Elodie D, Catherine F, Hadi Q (2011). Considering transposable element diversification in de novo annotation approaches. PLoS ONE.

[49] Chen N (2004). Using RepeatMasker to identify repetitive elements in genomic sequences. Curr. Protoc. Bioinformatics..

[50] Keilwagen J (2016). Using intron position conservation for homology-based gene prediction. Nucleic Acids Res..

[51] Nachtweide S, Stanke M (2019). Multi-genome annotation with AUGUSTUS. Methods Mol. Biol..

[52] Blanco E, Parra G, Guigó R (2018). Using geneid to identify genes. Curr. Protoc. Bioinform..

[53] Korf I (2004). Gene finding in novel genomes. BMC Bioinform..

[54] Majoros WH, Pertea M, Salzberg SL (2004). TigrScan and GlimmerHMM: two open source ab initio eukaryotic gene-finders. Bioinformatics.

[55] Kim D, Langmead B, Salzberg SL (2015). HISAT: a fast spliced aligner with low memory requirements. Nat. Methods.

[56] Pertea M (2015). StringTie enables improved reconstruction of a transcriptome from RNA-seq reads. Nat. Biotechnol..

[57] Kent WJ (2002). BLAT-the BLAST-like alignment tool. Genome Res..

[58] Haas BJ (2003). Improving the *Arabidopsis* genome annotation using maximal transcript alignment assemblies. Nucleic Acids Res..

[59] Grabherr MG (2011). Full-length transcriptome assembly from RNA-Seq data without a reference genome. Nat. Biotechnol..

[60] Tang S, Lomsadze A, Borodovsky M (2015). Identification of protein coding regions in RNA transcripts. Nucleic Acids Res..

[61] Haas BJ (2008). Automated eukaryotic gene structure annotation using EVidenceModeler and the program to assemble spliced alignments. Genome Biol..

[62] Camacho C (2009). BLAST+: architecture and applications. BMC Bioinform..

[63] Kanehisa M, Goto S, Kawashima S, Okuno Y, Hattori M (2004). The KEGG resource for deciphering the genome. Nucleic Acids Res..

[64] UniProt Consortium T. (2018). UniProt: the Universal Protein knowledgebase. Nucleic Acids Res..

[65] Deng Y (2006). Integrated nr Database in protein annotation system and its localization. Computer Eng..

[66] Conesa A (2005). Blast2GO: a universal tool for annotation, visualization and analysis in functional genomics research. Bioinformatics.

[67] Lowe TM, Chan PP (2016). tRNAscan-SE On-line: integrating search and context for analysis of transfer RNA genes. Nucleic Acids Res..

[68] Kozomara A, Griffithsjones S (2014). miRBase: annotating high confidence microRNAs using deep sequencing data. Nucleic Acids Res..

[69] Friedländer MR, Mackowiak SD, Li N, Chen W, Rajewsky N (2012). miRDeep2 accurately identifies known and hundreds of novel microRNA genes in seven animal clades. Nucleic Acids Res..

[70] Nawrocki EP, Eddy SR (2013). Infernal 1.1: 100-fold faster RNA homology searches. Bioinformatics.

[71] Gardner PP (2009). Rfam: updates to the RNA families database. Nucleic Acids Res..

[72] She R, Chu JS, Wang K, Pei J, Chen N (2009). GenBlastA: enabling BLAST to identify homologous gene sequences. Genome Res..

[73] Birney E, Durbin R (2000). Using GeneWise in the *Drosophila* annotation experiment. Genome Res.

[74] Li L, Stoeckert CJ, Roos DS (2003). OrthoMCL: identification of ortholog groups for eukaryotic genomes. Genome Res..

[75] Edgar RC (2004). MUSCLE: multiple sequence alignment with high accuracy and high throughput. Nucleic Acids Res..

[76] Guindon S (2010). New algorithms and methods to estimate maximum-likelihood phylogenies: assessing the performance of PhyML 3.0. Syst. Biol..

[77] Yang Z (2007). PAML 4: phylogenetic analysis by maximum likelihood. Mol. Biol. Evol..

[78] De Bie T, Cristianini N, Demuth JP, Hahn MW (2006). CAFE: a computational tool for the study of gene family evolution. Bioinformatics.

[79] Wang Y (2012). MCScanX: a toolkit for detection and evolutionary analysis of gene synteny and collinearity. Nucleic Acids Res..

[80] Hasegawa M, Kishino H, Yano T (1985). Dating of the human-ape splitting by a molecular clock of mitochondrial DNA. J. Mol. Evol..

[81] Sun S (2018). Extensive intraspecific gene order and gene structural variations between Mo17 and other maize genomes. Nat. Genet..

[82] Sønderby IE, Geu-Flores F, Halkier BA (2010). Biosynthesis of glucosinolates—gene discovery and beyond. Trends Plant Sci..

[83] Bednarek P (2009). A glucosinolate metabolism pathway in living plant cells mediates broad-spectrum antifungal defense. Science.

[84] Grubb CD, Abel S (2006). Glucosinolate metabolism and its control. Trends Plant Sci..

[85] Duan W (2014). Patterns of evolutionary conservation of ascorbic acid-related genes following whole-genome triplication in *Brassica rapa*. Genome Biol. Evol..

[86] Wang J (2017). Insights into the species-specific metabolic engineering of glucosinolates in radish (*Raphanus sativus* L.) based on comparative genomic analysis. Sci. Rep..

