# Supplementary material for: A chromosome-level reference genome of non-heading Chinese cabbage [Brassica campestris (syn. Brassica rapa) ssp. chinensis]
Source: Hortic Res. 2020 Dec 28;7:212. doi: 10.1038/s41438-020-00449-z (PMC7769993; doi:10.1038/s41438-020-00449-z)
Supplement: Supplementary file 1 — Supplementary Table S1-S17 [file 41438_2020_449_MOESM1_ESM.docx]

**Supplementary Table S1**  Statistics of the ONT, PacBio and Hi-C datasets. Standard metrics were computed using ONT reads (≥2000 bp) and PacBio reads (≥500 bp).

| Platform | Type | Read Bases (bp) | Reads Number | Read N50 (bp) | Mean Reads Length (bp) |
| --- | --- | --- | --- | --- | --- |
| Pacbio | Cleandata | 61,440,853,191 | 5,387,116 | 16,938 | 11,405 |
| Nanopore | Cleandata | 61,314,201,087 | 2,864,875 | 30,672 | 21,402 |
| Hi-C | Cleandata | 68,688,636,778 | 229,427,069 | - | - |

**Supplementary Table S2** Summary of the Hi-C assembly data.

| **Group** | **Cluster Num** | **Cluster Len(bp)** | **Order Num** | **Order Len(bp)** |
| --- | --- | --- | --- | --- |
| A01 | 74 | 41561427 | 46 | 38124912 |
| A02 | 21 | 35250367 | 11 | 33816594 |
| A03 | 36 | 43745763 | 22 | 41213565 |
| A04 | 20 | 24802955 | 14 | 23890470 |
| A05 | 68 | 45634568 | 45 | 42746886 |
| A06 | 113 | 49423914 | 62 | 43547417 |
| A07 | 28 | 32406715 | 9 | 29240466 |
| A08 | 7 | 25246887 | 3 | 23546502 |
| A09 | 153 | 73567804 | 72 | 64516467 |
| A10 | 27 | 25188622 | 16 | 23121081 |
| Total | 553(91.8%) | 396829022(97.9%) | 300(49.8%) | 363764360(89.7%) |

**Supplementary Table S3** Quality assessment of the assembled genome using BUSCO v4.0.6.

| Complete BUSCOs(C) | Complete and single-copy BUSCOs(S) | Complete and duplicated BUSCOs(D) | Fragmented BUSCOs(F) | Missing BUSCOs(M) | Total Lineage BUSCOs |
| --- | --- | --- | --- | --- | --- |
| 1599 (99.07%) | 1361  (84.32%) | 238  (14.75%) | 5  (0.31%) | 10  (0.62%) | 1614 |

**Supplemental Table S4** Error base percentage statistics for the *B. rapa* NHCC001 genome assembly.

| Contig_length(bp) | Correct_base_  number(bp) | Error_base_  number(bp) | Error_base_  percentage(%) |
| --- | --- | --- | --- |
| 405334585.00 | 405330102.00 | 4483.00 | 0.001106 |

**Supplemental Table S5** Genome completeness evaluated based on full-length transcripts.

| Range of length (bp) | Total number | Aligned number | | Percent (%) |
| --- | --- | --- | --- | --- |
| All | 35,031 | 34,811 | 99.37 | |
| >=300 | 34,711 | 34,494 | 99.37 | |
| >=1,000 | 27,324 | 27,149 | 99.36 | |

Note: only the full length transcripts with converage > 50% are considered.

**Supplementary Table S6** Quality assessment of the assembled genome and gene predictions using RNA-seq data.

| Sample | Total  Reads | Total_  Mapped | Unique Mapped  Reads | Mapped  Exon  Ratio | Mapped  Intro  Ratio | Mapped  Intergenic  Ratio |
| --- | --- | --- | --- | --- | --- | --- |
| Root-1 | 47,071,700 | 44,130,122 (93.75%) | 42,289,616 (89.84%) | 88.66% | 2.78% | 8.56% |
| Root-2 | 43,922,994 | 40,803,855 (92.90%) | 39,113,895 (89.05%) | 88.65% | 2.74% | 8.61% |
| Leaf-1 | 45,218,448 | 43,487,107 (96.17%) | 41,233,481 (91.19%) | 89.77% | 3.12% | 7.11% |
| Leaf-2 | 45,913,898 | 44,029,044 (95.89%) | 41,633,367 (90.68%) | 89.57% | 3.04% | 7.39% |

**Supplementary Table S7** Summary of the gene functional annotations in the *B. rapa* NHCC001 genome.

| Annotated Database | Annotated Number | 100<=Protein length<300 | Protein length>=300 |
| --- | --- | --- | --- |
| GO_Annotation | 35096 | 12117 | 21213 |
| KEGG_Annotation | 15201 | 5031 | 9489 |
| KOG_Annotation | 23672 | 7495 | 15189 |
| Pfam_Annotation | 35198 | 11279 | 22991 |
| Swissprot_Annotation | 31056 | 10018 | 19694 |
| TrEMBL_Annotation | 46965 | 17496 | 25390 |
| nr_Annotation | 45557 | 16943 | 24715 |
| nt_Annotation | 47215 | 17465 | 25340 |
| All_Annotated | 47872 | 17810 | 25400 |

**Supplementary Table S8** Statistics for TEs and repeats in the *B. rapa* NHCC001 genome.

| Type | Number | Length | Rate(%) |
| --- | --- | --- | --- |
| ClassI | 231196 | 167208723 | 41.25 |
| ClassI/DIRS | 10311 | 12483868 | 3.08 |
| ClassI/LARD | 98175 | 60873332 | 15.02 |
| ClassI/LINE | 29595 | 12986741 | 3.2 |
| ClassI/LTR/Copia | 27081 | 26078162 | 6.43 |
| ClassI/LTR/Gypsy | 46353 | 55086487 | 13.59 |
| ClassI/LTR/Unknown | 5227 | 1617168 | 0.4 |
| ClassI/PLE | 3670 | 2525794 | 0.62 |
| ClassI/SINE | 5599 | 1160622 | 0.29 |
| ClassI/TRIM | 3497 | 4490187 | 1.11 |
| ClassI/Unknown | 1688 | 565365 | 0.14 |
| ClassII | 47222 | 21760218 | 5.37 |
| ClassII/Crypton | 49 | 29591 | 0.01 |
| ClassII/Helitron | 2559 | 1044262 | 0.26 |
| ClassII/MITE | 2160 | 788784 | 0.19 |
| ClassII/Maverick | 1021 | 653729 | 0.16 |
| ClassII/TIR | 35097 | 16780279 | 4.14 |
| ClassII/Unknown | 6336 | 2822706 | 0.7 |
| PotentialHostGene | 15476 | 5608070 | 1.38 |
| SSR | 5170 | 1445614 | 0.36 |
| Unknown | 71086 | 33231750 | 8.2 |
| Total | 370150 | 213039551 | 52.56 |

**Supplementary Table S9** Statistics for gene families among 14 selected plant genomes.

| Name | Totalgene | oneCopynum | MultiCopynum | Unigenenum | Othergenenum | Clusternum | UnClusternum | Totalfamily | Unifamily |
| --- | --- | --- | --- | --- | --- | --- | --- | --- | --- |
| *B.rapa* NHCC001 | 48158 | 3256 | 17028 | 423 | 25609 | 46316 | 1842 | 27536 | 165 |
| *B.rapa* Chiifu | 46250 | 3442 | 15986 | 435 | 22677 | 42540 | 3710 | 25706 | 188 |
| *B.rapa* Z1 | 46721 | 3506 | 15425 | 641 | 21581 | 41153 | 5568 | 25255 | 243 |
| *C.rubella* | 26521 | 5417 | 8105 | 294 | 10835 | 24651 | 1870 | 16905 | 112 |
| *C.papaya* | 18003 | 6282 | 4479 | 538 | 3582 | 14881 | 3122 | 11156 | 193 |
| *A.thaliana* | 27416 | 5401 | 8139 | 168 | 11490 | 25198 | 2218 | 17780 | 70 |
| *C.hirsuta* | 29458 | 5426 | 8141 | 333 | 10591 | 24491 | 4967 | 17078 | 133 |
| *S.parvula* | 28901 | 4726 | 10050 | 1588 | 10206 | 26570 | 2331 | 15943 | 495 |
| *B.nigra* | 47953 | 3482 | 16112 | 2052 | 15998 | 37644 | 10309 | 19815 | 787 |
| *L.alabamica* | 38676 | 4368 | 12818 | 4025 | 10390 | 31601 | 7075 | 15379 | 883 |
| *T.halophila* | 26351 | 5456 | 7781 | 434 | 10435 | 24106 | 2245 | 16478 | 130 |
| *B.oleracea* | 45758 | 3527 | 15394 | 1451 | 16551 | 36923 | 8835 | 20972 | 551 |
| *A.lyrata* | 32670 | 5305 | 8452 | 2062 | 11853 | 27672 | 4998 | 18240 | 613 |
| *A.arabicum* | 37839 | 5729 | 8140 | 5605 | 12167 | 31641 | 6198 | 14441 | 973 |

**Supplementary Table S10** Statistics for SNPs and indels identified between *B. rapa* NHCC001, *B. rapa* Chiifu, and *B. rapa* Z1.

|  |  | Number | Length |
| --- | --- | --- | --- |
| Brapa.Z1_NHCC001 | SNP | 1718037 | 1718037 |
|  | INDEL | 738275 | 2,721,123 |
| Brapa.chiifu_NHCC001 | SNP | 1305874 | 1305874 |
|  | INDEL | 469629 | 1981969 |

**Supplementary Table S11** Summary of PAV sequences between the *B. rapa* NNHCC001 and *B. rapa* Z1 genomes and between the *B. rapa* NNHCC001 and *B. rapa* Chiifu genomes.

|  |  | *B. rapa* Chiifu_NHCC001 | | *B. rapa* Z1_NHCC001 | |
| --- | --- | --- | --- | --- | --- |
|  |  | Chiifu-specific | NHCC001-specific | Z1-specific | NHCC001-specific |
| Total | Number | 8496 | 10851 | 12744 | 13524 |
|  | Length | 10169926 | 13051872 | 15667128 | 16480280 |
|  | Gene number | 1979 | 2534 | 2926 | 3102 |
| 500-1k(<1k) | Number | 5156 | 6594 | 7875 | 8101 |
|  | Length | 3161815 | 4054711 | 4811761 | 4984101 |
| 1-2k(>=1k ,<2k) | Number | 2130 | 2694 | 3013 | 3352 |
|  | Length | 2804400 | 3597183 | 3984758 | 4454001 |
| 2-3k | Number | 576 | 756 | 879 | 1063 |
|  | length | 1367449 | 1815600 | 2099297 | 2530600 |
| 3-4k | Number | 279 | 352 | 393 | 427 |
|  | length | 936400 | 1175200 | 1325931 | 1438500 |
| 4-5k | Number | 178 | 233 | 256 | 287 |
|  | length | 792500 | 1028500 | 1135760 | 1273900 |
| >=5k | Number | 177 | 222 | 328 | 294 |
|  | length | 1107362 | 1380678 | 2309621 | 1799178 |
| Total Gene number | | 2534 | 1979 | 2926 | 3102 |

**Supplementary Table S12** Summary of PAV clusters longer than 500 kb between the *B. rapa* NNHCC001 and *B. rapa* Chiifu genomes.

|  | Region | Size(bp) | Gene |
| --- | --- | --- | --- |
|  |  |  | number |
| PAVs cluster in NHCC001 | A02:7363101-7896300 | 533200 | 50 |
|  | A07:4154001-4773300 | 619300 | 41 |
|  | A07:9301901-9845200 | 543300 | 41 |
| PAVs cluster in Chiifu | A07:4026801-4566800 | 540000 | 50 |
|  | A08:1930601-2481100 | 550500 | 79 |

**Supplementary Table S13** Summary of PAV clusters longer than 500 kb between the *B. rapa* NNHCC001 and *B. rapa* Z1 genomes.

|  | **Region** | **Size(bp)** | **Gene** |
| --- | --- | --- | --- |
|  |  |  | **number** |
| PAVs cluster in NHCC001 | A02:7336901-8001800 | 664900 | 68 |
|  | A03:21340401-21895600 | 555200 | 92 |
|  | A04:19412001-19952000 | 540000 | 90 |
|  | A06:12826601-13484900 | 658300 | 59 |
|  | A07:4244301-4825900 | 581600 | 39 |
|  | A08:19584001-20126500 | 542500 | 94 |
|  | A09:13848101-14355600 | 507500 | 47 |
|  | A09:5775101-6481300 | 706200 | 107 |
| PAVs cluster in Z1 | A02:3046301-3571300 | 525000 | 122 |
|  | A09:12747501-13288600 | 541100 | 63 |

**Supplementary Table S14** Leaf adaxial-abaxial patterning genes reported in *A. thaliana* and their homologs identified in *B. rapa* NHCC001, *B. rapa* Chiifu, and *B. rapa* Z1.

| **Adaxial determinants** | **Gene** | ***Arabidopsis thaliana*** | ***B.rapa NHCC001*** | ***B.rapa Chiifu*** | ***B.rapa* Z1** |
| --- | --- | --- | --- | --- | --- |
| **HD-ZIP Ⅲ** | **REV** | **AT5G60690** | **BraC10g019890/BraC02g010360/BraC06g021780** | **BraA10g018460.3C/BraA02g010200.3C/BraA06g021080.3C** | **BraA10t43868Z/BraA02t05711Z/BraA06t25241Z** |
|  | **PHB** | **AT2G34710** | **BraC04g026800/BraC05g010550** | **BraA04g024760.3C/BraA05g010360.3C** | **BraA05t20039Z/BraA04t18157Z** |
|  | **PHV** | **AT1G30490** | **BraC09g040010** | **BraA09g034560.3C** | **BraA09t39121Z** |
|  | **ATHB8** | **AT4G32880** | **BraC01g005070/BraC08g017630** | **BraA01g005160.3C/BraA08g016820.3C** | **BraA01t00514Z/BraA08t33735Z** |
| **ARP** | **AS1** | **AT2G37630** | **BraC03g019910/BraC08g017630** | **BraA03g019630.3C/BraA05g007920.3C** | **BraA03t11318Z/BraA05t19798Z** |
| **LOB** | **AS2** | **AT1G65620** | **BraC02g017320** | **BraA02g016770.3C** | **BraA02t06386Z** |
| **Middle domain determinants** |  |  |  |  |  |
| **YABBY** | **YAB1** | **AT2G45190** | **BraC03g023950/BraC04g033560/BraC07g023070** | **BraA03g023630.3C/BraA04g030760.3C/BraA07g023150.3C** | **BraA03t11711Z/BraA04t18831Z/BraA07t30030Z** |
|  | **YAB2** | **AT1G08465** | **BraC08g034030/BraC06g005490/BraC09g070760** | **BraA06g005620.3C/BraA08g033150.3C/BraA09g062760.3C** | **BraA06t23754Z/BraA08t35422Z/BraA09t41932Z** |
|  | **YAB3** | **AT4G00180** | **BraC09g001620** | **-** | **BraA09t35690Z** |
|  | **YAB5** | **AT2G26580** | **BraC03g025740** | **BraA03g025390.3C** | **BraA03t11900Z** |
| **WOX** | **WOX1** | **AT3G18010** | **BraC03g038810/BraC05g035880** | **BraA05g029540.3C/BraA03g038230.3C** | **BraA05t22013Z/BraA03t13208Z** |
|  | **WOX3** | **AT2G28610** | **BraC04g022620/BraC03g025160** | **BraA04g020450.3C/BraA03g024810.3C** | **BraA04t17732Z/BraA03t11842Z** |
| **Abaxial determinants** |  |  |  |  |  |
| **KANDAI** | **KAN1** | **AT5G16560** | **BraC10g024720/BraC02g006720/BraC03g007660** | **BraA02g006540.3C/BraA03g007570.3C/BraA10g023200.3C** | **BraA10t44326Z/BraA02t05332Z/BraA03t10110Z** |
|  | **KAN2** | **AT1G32240** | **BraC05g029280/BraC09g037900/BraC05g028580** | **BraA05g023490.3C/BraA09g032840.3C** | **BraA05t21391Z/BraA09t38937Z** |
|  | **KAN3** | **AT4G17695** | **BraC08g013450/BraC01g009250** | **BraA08g012540.3C/BraA01g009320.3C** | **BraA08t33310Z/BraA01t00921Z** |
| **ARF** | **ARF3** | **AT2G33860** | **BraC05g011270/BraC04g026370** | **BraA05g011080.3C/BraA04g024390.3C** | **BraA04t18111Z/BraA05t20115Z** |
|  | **ARF4** | **AT5G60450** | **BraC10g019130/BraC02g010480/BraC02g010500** | **BraA02g010280.3C/BraA10g018230.3C** | **BraA02t05722Z/BraA02t05724Z/BraA10t43845Z** |
| **Generators of small RNAs** |  |  |  |  |  |
| **AGO** | **AGO1** | **AT1G48410** | **BraC08g004770/BraC05g021470** | **BraA08g004410.3C/BraA05g020200.3C** | **BraA08t32502Z/BraA05t21010Z** |
|  | **AGO7** | **AT1G69440** | **BraC07g030310/BraC02g019920** | **BraA07g030370.3C** | **BraA02t06644Z/BraA07t30789Z** |
|  | **AGO10** | **AT5G43810** | **BraC09g019420/BraC06g050750** | **BraA09g019960.3C/BraA06g043560.3C** | **BraA06t27637Z/BraA09t37661Z** |
| **SGS** | **SGS3** | **AT5G23570** | **BraC06g037620** | **BraA06g031000.3C** | **BraA06t26381Z** |
| **RDR** | **RDR6** | **AT3G49500** | **BraC01g025260/BraCxxg005900** | **BraA01g023640.3C** | **BraA01t02383Z** |
| **HYL** | **HYL1** | **AT1G09700** | **BraC08g033350/BraC06g006460** | **BraA08g032470.3C/BraA06g006590.3C** | **BraA08t35352Z/BraA06t23849Z** |
| **DCL** | **DCL1** | **AT1G01040** | **BraC10g000840** | **BraA10g000850.3C/BraA10g000840.3C** | **BraA10t42365Z** |
|  | **DCL4** | **AT5G20320** | **BraC10g021710** | **BraA10g020250.3C** | **BraA10t44037Z** |
| **SE** | **SE** | **AT2G27100** | **BraC04g021620/BraC07g017830** | **BraA04g019460.3C/BraA07g018000.3C** | **BraA04t17633Z/BraA07t29518Z** |

**Supplementary Table S15** Ascorbic acid-related genes reported in *A. thaliana* and homologs identified in *B. rapa* NHCC001, *B. rapa* Chiifu, and *B. rapa* Z1.

|  | **Genes** | ***A.thaliana*** | ***B. rapa* NHCC001** | ***B. rapa* Chiifu** | ***B. rapa* Z1** |
| --- | --- | --- | --- | --- | --- |
| **L-Galactose pathway** | **PGI** | **AT4G24620** | **BraC01g015750.1/**  **BraC03g053670.1** | **BraA01g015390.3C/**  **BraA03g052000.3C** | **BraA01t01531Z/**  **BraA03t14618Z** |
|  |  | **AT5G42740** | **BraC09g018780.1** | **BraA09g019380.3C** | **BraA09t37600Z** |
|  | **PMI** | **AT1G67070** | **BraC02g017950.1** | **BraA02g017380.3C** | **BraA02t06451Z** |
|  |  | **AT3G02570** | **BraC05g048740.1/**  **BraC01g049190.1** | **BraA05g041300.3C/**  **BraA01g044070.3C** | **BraA05t23135Z/**  **BraSC246t45833Z** |
|  | **PMM** | **AT2G45790** | **BraC03g024280.1/**  **BraC04g033900.1/**  **BraC05g005780.1** | **BraA03g023960.3C/**  **BraA05g005470.3C/**  **BraA04g031750.3C** | **BraA04t18865Z/**  **BraA03t11743Z/**  **BraA05t19544Z** |
|  | **GMPase** | **AT1G74910** | **BraC02g023380.1/**  **BraC07g028090.1/**  **BraC07g038620.1/** | **BraA02g022530.3C/**  **BraA07g028050.3C/**  **BraA07g038620.3C** | **BraA02t06997Z/**  **BraA07t31599Z/**  **BraA07t30557Z** |
|  |  | **AT2G39770** | **BraC03g021350.1/**  **BraC04g029970.1/**  **BraC05g006630.1** | **BraA04g027980.3C/**  **BraA03g021070.3C/**  **BraA05g006280.3C** | **BraA04t18482Z/**  **BraA03t11460Z/**  **BraA05t19629Z** |
|  |  | **AT3G55590** |  |  |  |
|  |  | **AT4G30570** | **BraC03g058250.1/**  **BraC03g058160.1/**  **BraC03g027860.1** | **BraA03g055960.3C/**  **BraA03g055870.3C/**  **BraA03g027370.3C** | **BraA03t15053Z/**  **BraA03t12105Z** |
|  | **GME** | **AT5G28840** | **BraC06g041090.1/**  **BraC02g042580.1** | **BraA06g034520.3C/**  **BraA02g040380.3C** | **BraA02t08808Z/**  **BraA06t26724Z** |
|  | **GGalPP** | **AT4G26850** | **BraC08g020580.1/**  **BraC03g055170.1/**  **BraC01g017980.1** | **BraA01g017580.3C/BraA08g019700.3C/BraA03g053380.3C** | **BraA01t01762Z/**  **BraA08t34022Z/**  **BraA03t14766Z** |
|  |  | **AT5G55120** |  | **BraA02g013520.3C** | **BraA02t06052Z** |
|  | **GalPP** | **AT3G02870** | **BraC05g048650.1/**  **BraC03g031690.1** | **BraA05g041500.3C/BraA03g031190.3C** | **BraA06t23281Z/**  **BraA03t12488Z** |
|  | **GalDH** | **AT4G33670** | **BraC03g060710.1/**  **BraC08g017250.1** | **BraA03g058040.3C/BraA08g016480.3C** | **BraA03t15255Z/**  **BraA08t33702Z** |
|  | **GalLDH** | **AT3G47930** | **BraC06g019160.1** | **BraA06g019070.3C** | **BraA06t25053Z** |
| **Galacturonate pathway** | **GalUR** | **AT1G59950** | **BraC09g016560.1** | **BraA09g017490.3C** | **BraA09t37415Z/BraA05t19316Z** |
|  |  | **AT1G59960** |  |  |  |
|  |  | **AT2G37760** | **BraC05g008100.1/**  **BraC05g008120.1/**  **BraC05g008130.1/**  **BraC03g019970.1/**  **BraC03g019980.1/**  **BraC05g008150.1** | **BraA05g007790.3C/BraA05g007780.3C/BraA05g007770.3C/BraA03g019700.3C/BraA03g019710.3C/BraA05g007750.3C** | **BraA03t11325Z/BraA03t11326Z/BraA05t19781Z/BraA05t19782Z/BraA05t19784Z/BraA05t19785Z/BraA05t19786Z** |
|  |  | **AT2G37770** |  |  |  |
|  |  | **AT2G37790** |  |  |  |
|  |  | **AT5G62420** | **BraC02g046070.1/BraC06g031790.1** | **BraA06g025940.3C/BraA02g043670.3C** | **BraA06t25860Z/BraA02t09158Z** |
|  |  | **AT5G01670** | **BraC03g000470.1** | **BraA03g000210.3C** | **BraA03t09405Z** |
|  |  | **AT3G53880** | **BraC09g052410.1** | **BraA09g044840.3C** | **BraA09t40151Z** |
|  | **Alase** | **AT3G03060** | **BraC01g048810.1/BraC05g048390.1** | **BraA01g043880.3C/BraA05g041610.3C** | **BraA01t04596Z/BraSC90t46699** |
|  |  | **AT5G16930** | **BraC02g006940.1/BraC10g024370.1** | **BraA02g006750.3C/BraA10g022890.3C** | **BraA10t44291Z/BraA02t05352Z** |
|  |  | **AT2G18330** | **BraC06g036250.1/BraC09g010700.1** | **BraA09g011360.3C/BraA06g029880.3C** | **BraA09t36829Z/BraA06t26257Z** |
|  |  | **AT4G36580** | **BraC01g002160.1** | **BraA01g002210.3C** | **BraA01t00219Z/** |
| **L-Gulose pathway** | **GLOase** | **AT2G46740** | **BraC05g001520.1/BraC05g001530.1** | **BraA05g001100.3C/BraA05g001110.3CBraA05g001120.3C** | **BraA05t19105Z/BraA05t19106Z/BraA05t19104Z** |
|  |  | **AT2G46750** |  |  |  |
|  |  | **AT2G46760** |  |  |  |
|  |  | **AT5G11540** | **BraC02g004120.1** | **BraA02g003980.3C** | **BraA02t05085Z** |
|  |  | **AT1G32300** |  |  |  |
|  |  | **AT5G56490** | **BraC02g013070.1/BraC10g014880.1/BraC10g014870.1** | **BraA02g012830.3C/BraA10g014340.3C** | **BraA02t05974Z/BraA10t43476Z/BraA10t43475Z** |
|  |  | **AT5G56470** |  |  |  |
| ***myo*-Inositol pathway** | **IPS** | **AT4G39800** | **BraC01g000740.1** | **BraA01g000700.3C** | **BraA01t00076Z** |
|  |  | **AT2G22240** | **BraC09g063010.1/BraC04g018280.1** | **BraA09g055030.3C/BraA04g016220.3C** | **BraA04t17307Z/BraA09t41162Z** |
|  |  | **AT5G10170** | **BraC02g003440.1/BraC10g029290.1** | **BraA02g003370.3C/BraA10g027710.3C** | **BraA02t05022Z/BraA10t44789Z** |
|  | **MIOX** | **AT1G14520** | **BraC06g010380.1** | **BraA06g010690.3C** | **BraA06t24237Z** |
|  |  | **AT2G19800** | **BraC07g001040.1/BraC09g011440.1** | **BraA07g001030.3C/BraA09g012070.3C** | **BraA07t27891Z/BraA09t36906Z** |
|  |  | **AT4G26260** | **BraC03g054830.1** | **BraA03g053010.3C** | **BraA03t14727Z** |
|  |  | **AT5G56640** | **BraC02g012980.1** | **BraA02g012730.3C** | **BraA02t05966Z** |
| **Recycling pathway** | **AO** | **AT5G21100** | **BraC10g021110.1/BraC10g021130.1/BraC02g009420.1/BraC02g009430.1** | **BraA02g009330.3C/BraA02g009340.3C/BraA10g019580.3C/BraA10g019570.3C/BraA10g019590.3C** | **BraA02t05614Z/BraA02t05615Z/BraA10t43974Z/BraA10t43975Z/BraA10t43976Z** |
|  |  | **AT5G21105** |  |  |  |
|  |  | **AT4G39830** | **BraC01g039760.1** | **BraA01g035310.3C** | **BraA01t03759Z** |
|  | **APX** | **AT1G07890** | **BraC09g071060.1/BraC06g004980.1/BraC08g034310.1** | **BraA09g063050.3C/BraA06g005140.3C/BraA08g033370.3C** | **BraA06t23706Z/BraA08t35447Z/BraA09t41951Z** |
|  |  | **AT3G09640** | **BraC01g045650.1** | **BraA01g040660.3C** | **BraA01t04291Z** |
|  |  | **AT4G35000** | **BraC03g062100.1** | **BraA03g059120.3C/BraA03g043300.3C** | **BraA03t15399Z/BraA03t13728Z** |
|  |  | **AT4G35970** | **BraC01g002570.1** | **BraA01g002610.3C** | **BraA01t00260Z** |
|  |  | **AT4G32320** | **BraC03g059550.1** | **BraA03g057260.3C** | **BraA03t15178Z** |
|  |  | **AT4G09010** | **BraC03g044730.1** | **BraA03g026770.3C** | **BraA03t12039Z** |
|  |  | **AT4G08390** | **BraC09g029270.1** | **BraA09g029180.3C** | **BraA09t38586Z** |
|  |  | **AT1G77490** | **BraC07g040820.1** | **BraA07g040850.3C** | **BraA07t31815Z** |
|  | **MDAR** | **AT5G03630** | **BraC03g001520.1** | **BraA03g001240.3C** | **BraA03t09511Z** |
|  |  | **AT3G09940** | **-** | **BraA03g033690.3C** | **BraA03t12749Z** |
|  |  | **AT3G52880** | **BraC09g051560.1** | **BraA09g043930.3C** | **BraA09t40055Z** |
|  |  | **AT3G27820** | **BraC06g044350.1/BraC02g040180.1** | **BraA06g037360.3C/BraA02g037920.3C** | **BraA06t27010Z/BraA02t08567Z** |
|  |  | **AT1G63940** | **BraC09g012760.1** | **BraA09g013500.3C** | **BraA09t37050Z** |
|  | **DHAR** | **AT1G19570** | **BraC06g014730.1/BraC08g029030.1** | **BraA06g015150.3C/BraA08g028190.3C** | **BraA06t24683Z** |
|  |  | **AT1G19550** |  |  |  |
|  |  | **AT1G75270** | **BraC02g023560.1** | **BraA02g022700.3C** | **BraA02t07017Z** |
|  |  | **AT5G16710** | **BraC10g024570.1/BraC02g006800.1** | **BraA02g006620.3C** | **BraA10t44311Z** |

Note：The colored blue genes refer to occur tandem duplication

**Supplementary Table S16**. GSL genes reported in *A. thaliana* and homologs identified in *B. rapa* NHCC001, *B. rapa* Chiifu, and *B. rapa* Z1.

|  | **Genes** | ***Arabidopsis thaliana*** | ***B. rapa* NHCC001** | ***B. rapa* Chiifu** | ***B. rapa* Z1** |
| --- | --- | --- | --- | --- | --- |
| Transcription factors | OBP2 | AT1G07640.3 | BraC08g034440.1 | BraA08t35458Z | BraA08g033480.3C |
|  | IQD1 | AT3G09710.2 | BraC01g045610.1 | BraA01t04287Z | BraA01g040610.3C |
|  | MYB28 | AT5G61420.2 | BraC03g045680.1 | BraA09t36383Z | BraA03g044380.3C |
|  |  |  | BraC09g006320.1 | BraA03t13824Z | BraA09g006980.3C |
|  |  |  | BraC02g045540.1 | BraA02t09109Z | BraA02g043150.3C |
|  |  |  | BraC02g045350.1 | BraA10t44983Z |  |
|  | MYB29 | AT5G07690.1 | BraC10g031440.1 | BraA10t44983Z | BraA10g029630.3C |
|  |  |  | BraC03g003410.1 | BraA03t09692Z | BraA03g003120.3C |
|  | MYB34 | AT5G60890.1 | BraC03g045270.1 | BraA03t13782Z | BraA03g043830.3C |
|  |  |  | BraC09g006090.1 | BraA09t36361Z | BraA09g006740.3C |
|  |  |  | BraC02g045040.1 | BraA02t09069Z | BraA02g042740.3C |
|  |  |  |  | BraA02t09070Z | BraA02g042730.3C |
|  | MYB51 | AT1G18570.1 | BraC08g029560.1 | BraA08t34968Z | BraA08g028700.3C |
|  |  |  | BraC06g013880.1 | BraA06t24589Z | BraA06g014200.3C |
|  |  |  | BraC09g065110.1 | BraA09t41359Z | BraA09g057180.3C |
|  | MYB76 | AT5G07700.1 | -- |  |  |
|  | MYB115 | AT5G40360.1 | -- |  |  |
|  | MYB118 | AT3G27785.1 | BraC02g040170.1 | BraA02t08566Z | BraA06g037390.3C |
|  |  |  | BraC06g044390.1 | BraA06t27014Z | BraA02g037910.3C |
|  | MYB122 | AT1G74080.1 | BraC02g022990.1 | BraA02t06956Z | BraA07g038040.3C |
|  |  |  | BraC07g038050.1 | BraA07t31540Z | BraA02g022090.3C |
| Side-chain elongation | BCAT3 | AT3G49680.1 | BraC06g016990.1 | BraA06t24893Z | BraA06g017480.3C |
|  |  |  | BraC06g017340.2 | BraA01t02371Z | BraA01g023540.3C |
|  |  |  | BraC01g025190.1 | BraA06t23889Z | BraA06g006960.3C |
|  |  |  | BraC01g025160.1 |  |  |
|  | **BCAT4** | AT3G19710.1 | BraC05g033720.1  BraC05g034010.1  BraC03g039990.1  BraC03g039570.1 | BraA05t21834Z  BraA03t13292Z | BraA05g027600.3C  BraA03g039020.3C |
|  | BAT5 | AT4G12030.2 | BraC03g028340.1 | BraA03t12148Z | BraA03g027780.3C |
|  |  |  | BraC09g025920.1 | BraA09t38280Z | BraA09g026540.3C |
|  | IPMI-LSU1 | AT4G13430.1 | BraC08g007040.1 | BraA08t32699Z | BraA08g006460.3C |
|  |  |  | BraC04g009210.1 | BraA04t16563Z | BraA04g008640.3C |
|  | IPMI-SSU2 | AT2G43100.1 | BraC05g003780.1/BraC05g003770.1 | BraA05t19345Z/BraA05t19344Z | BraA05g003390.3C/BraA05g003380.3C |
|  |  |  |  |  |  |
|  | IPMI-SSU3 | AT3G58990.1 |  |  |  |
|  |  |  |  |  |  |
|  | IPMDH1 | AT5G14200.1 | BraC02g005440.1 BraC02g026910.1 BraC07g042860.1BraC02g026920.1 BraC02g026940.1 | BraA02t05207Z BraA02t07337Z BraA07t32014Z BraA02t07338Z BraA02t07340Z | BraA02g005320.3C BraA02g025810.3C BraA07g042910.3C BraA02g025820.3C BraA02g025840.3C |
|  | IPMDH3 | AT1G31180.1 |  |  |  |
|  | MAM1 | AT5G23010.1 | BraC02g044960.1/BraC02g044970.1/BraC03g045180.1/BraC03g045190.1/BraC03g045200.1/BraC02g028600.1/BraC04g027040.1 | BraA03t13775Z/BraA03t13773Z/BraA02t07492Z/BraA02t07413Z/BraA02t09063Z | BraA02g042670.3C/BraA02g042660.3C/BraA03g043740.3C/BraA03g043750.3C/BraA03g043730.3C/BraA02g027350.3C/BraA02g026580.3C/BraA04g024970.3C/BraA04g024980.3C |
|  | MAM3 | AT5G23020.1 |  |  |  |
|  |  |  |  |  |  |
|  |  |  |  |  |  |
| Core structure formation | CYP79A2 | AT5G05260.1 | BraC02g001680.1 | BraA02t04840Z | BraA02g001640.3C |
|  |  |  | BraC10g033010.1 | BraA10t45138Z | BraA10g028190.3C |
|  | CYP79B2 | AT4G39950.1 | BraC01g000830.1 | BraA03t15631Z | BraA01g000830.3C |
|  |  |  | BraC03g064620.1 | BraA01t00085Z | BraA03g061220.3C |
|  |  |  | BraC08g022740.1 |  | BraA08g022020.3C |
|  | CYP79B3 | AT2G22330.1 | BraC04g018420.1 | BraA04t17315Z | BraA04g016310.3C |
|  |  |  | BraC01g000830.1 | BraA01t00085Z | BraA01g000830.3C |
|  | CYP79F1 | AT1G16410.1 | BraC06g012030.1 | BraA06t24399Z | BraA06g012350.3C |
|  | CYP79F2 | AT1G16400.1 |  |  |  |
|  | CYP83A1 | AT4G13770.1 | BraC04g031760.1 | BraA04t18644Z | BraA04g029510.3C |
|  |  |  | BraC04g008890.1 | BraA04t16535Z | BraA04g008320.3C |
|  | CYP83B1 | AT4G31500.1 | BraC08g008210.1 | BraA08t32795Z | BraA08g007500.3C |
|  | GSTF9 | AT2G30860.1 | BraC04g024270.1 | BraA03t10995Z | BraA03g016420.3C |
|  |  |  | BraC03g016710.1 | BraA04t17883Z | BraA04g022100.3C |
|  | GSTF10 | AT2G30870.1 | BraC03g016720.1 | BraA03t10996Z | BraA03g016430.3C |
|  | GSTF11 | AT3G03190.1 | BraC05g048290.1 | BraSC90t46710Z | BraA05g041710.3C |
|  | GSTU20 | AT1G78370.1 | BraC07g026730 | BraA07t30399Z | BraA07g026620.3C |
|  | GGP1 | AT4G30530.1 | BraC08g019000.1 | BraA01t00708Z | BraA08g018030.3C |
|  |  |  | BraC01g007140.1 | BraA08t33880Z | BraA01g007240.3C |
|  |  |  | BraC03g058140.1 | BraA03t15051Z | BraA03g055850.3C |
|  | SUR1 | AT2G20610.1 | BraC07g000220.1 | BraA07t27807Z | BraA07g000220.3C |
|  |  |  | BraC09g011420.1 | BraA09t36904Z | BraA09g012050.3C |
|  | UGT74B1 | AT1G24100.1 | BraC09g045630.1 | BraA09t39573Z | BraA09g039260.3C |
|  | UGT74C1 | AT2G31790.1 | BraC05g013320.1 | BraA05t20311Z | BraA05g012890.3C |
|  |  |  | BraC04g025030.1 | BraA04t17962Z | BraA04g022880.3C |
|  | ST5a | AT1G74100.1 | BraC07g038090.1/BraC07g038080.1/BraC02g023030.1/BraC07g038060.1/BraC09g017150.1/BraC09g017390.1/BraC09g012270.1BraC09g017190.1/BraC09g017450.1/BraC09g015810.1/BraC09g015800.1/BraC07g027520.1/BraC09g017170.1/BraC09g017420.1 | BraA02t06957Z/BraA07t31543Z/BraA07t31541Z/BraA07t30593Z/BraA07t30594Z/BraA09t36999Z/BraA09t37474Z/BraA09t37341Z/BraA09t37340Z | BraA07g038080.3C/BraA07g038070.3C/BraA02g022100.3C/BraA07g038050.3C/BraA07g028440.3C/BraA07g028430.3C/BraA09g018130.3C/BraA09g018070.3C/BraA09g012950.3C/BraA09g016690.3C/BraA09g016680.3C/BraA07g027460.3C/BraA01g028570.3C/BraA09g018100.3C |
|  |  |  |  |  |  |
|  |  |  |  |  |  |
|  | ST5b | AT1G74090.1 |  |  |  |
|  |  |  |  |  |  |
|  |  |  |  |  |  |
|  | ST5c | AT1G18590.1 | BraC06g013900.1 | BraA06t24591Z | BraA06g014220.3C |
| Secondary modification | FMOGS-OX1 | AT1G65860.1 | BraC09g014670.1/BraC08g032000.1/BraC09g014640.2/BraC09g014630.1/BraC09g068930.1/BraC09g068950.1 | BraA09t37255Z/BraA09t41753Z/BraA08t35217Z | BraA09g015770.3C/BraA08g031160.3C/BraA09g060940.3C |
|  | FMOGS-OX2 | AT1G62540.1 |  |  |  |
|  | FMOGS-OX3 | AT1G62560.1 |  |  |  |
|  | FMOGS-OX4 | AT1G62570.1 |  |  |  |
|  | FMOGS-OX5 | AT1G12140.1 |  |  |  |
|  |  |  |  |  |  |
|  |  |  |  |  |  |
|  |  |  |  |  |  |
|  | AOP1 | AT4G03070.1 | BraCxxg009730.1/BraC02g028650.1/BraC03g029330.1/BraC03g029320.1/ BraCxxg009700.1/BraCxxg009710.1 | BraA02t07494Z/ BraA03t12246Z/ BraA03t12244Z/ BraA09t35886Z/ BraA09t35885Z/BraA09t35884Z | BraA03g028730.3C/BraA03g028740.3C/BraA09g001270.3C/BraA09g001280.3C/BraA09g001290.3C/BraA02g027370.3C |
|  | AOP3 | AT4G03050.2 |  |  |  |
|  | AOP2 | AT4G03060.1 |  |  |  |
|  | GSL-OH | AT2G25450.1 | BraC03g017760.1 | BraA03t11110Z | BraA03g017510.3C |
|  |  |  | BraC04g024240.1 | BraA04t17882Z | BraA04g022060.3C |
|  |  |  | BraC04g024230.1 | BraA04t17879Z | BraA04g022080.3C |
|  |  |  | BraC04g024220.1 | BraA04t17880Z | BraA04g022070.3C |
|  |  |  |  |  | BraA04g022090.3C |
|  | CYP81F2 | AT5G57220.1 | BraC02g012690.1 | BraA02t05935Z | BraA02g012430.3C |
|  |  |  | BraC10g015730.1 | BraA10t43550Z | BraA10g015140.3C |
|  |  |  | BraC03g012730.1 | BraA03t10602Z | BraA03g012530.3C |
|  | CYP81F3 | AT4G37400.1 | BraC01g001510.1 | BraA08t34226Z | BraA01g001490.3C |
|  |  |  | BraC08g022290.1 | BraA01t00153Z | BraA08g021490.3C |
|  | CYP81F1 | AT4G37430.1 | BraC01g001470.1 | BraA01t00149Z | BraA01g001460.3C |
|  |  |  | BraC01g001480.1 | BraA01t00150Z | BraA01g001470.3C |
|  | CYP81F4 | AT4G37410.1 | BraC08g022300.1 | BraA08t34230Z | BraA01g001490.3C |
|  |  |  | BraC01g001500.1 | BraA01t00152Z | BraA08g021500.3C |
| Co-substrate pathway | BZO1 | AT1G65880.1 | BraC06g024450.1 | BraA07t30928Z | BraA06g022640.3C |
|  |  |  | BraC02g017430.1 | BraA02t06398Z | BraA07g031810.3C |
|  |  |  |  | BraA06t25396Z | BraA02g016850.3C |
|  | APK1 | AT2G14750.1 | BraC09g009460.1 | BraA09t36694Z | BraA09g010100.3C |
|  |  |  | BraC03g044060.1 | BraA03t13660Z | BraA03g042630.3C |
|  | APK2 | AT4G39940.1 | BraC01g000820.1 | BraA01t00084Z | BraA01g000790.3C |
|  |  |  | BraC03g064630.1 | BraA03t15632Z | BraA03g061240.3C |
|  |  |  | BraC08g022750.1 | BraA08t34277Z | BraA08g022030.3C |
|  | CHY1 | AT5G65940.1 | BraC02g048050.2 | BraA02t09380Z | BraA02g045840.3C |
|  | GSH1_PAD2 | AT4G23100.1 | BraC03g052340.1 | BraA01t01385Z | BraA01g013720.3C |
|  |  |  | BraC01g014100.1 | BraA03t14471Z | BraA03g050750.3C |
|  |  |  | BraC03g052330.1 | BraA03t14470Z | BraA03g050740.3C |
|  | AAO4 | AT1G04580.1 | BraC10g003050.1 | BraA10t42570Z | BraA10g003130.3C |
| Breakdown pathway | TGG1 | AT5G26000.1 | BraC09g009530.1/BraC02g043630.1/BraC02g043340.1/BraC02g043360.1/BraC09g040630.1/BraC07g030450.1/BraC08g011810.1/BraC08g030860.1/BraC08g011870.1/BraC01g035680.1/BraC02g043320.1 | BraA09t36700Z/BraA09t36702Z/BraA07t30802Z/BraA08t35098Z**/**BraA02t08914Z/BraA02t08882Z/BraA02t08880Z/BraA08t33148Z/BraA08t33158Z/**/**BraA08t33155Z/BraA03t12106Z/BraA02t08878Z |  |
|  |  |  |  |  |  |
|  |  |  |  |  |  |
|  |  |  |  |  |  |
|  |  |  |  |  |  |
|  |  |  |  |  |  |
|  |  |  |  |  |  |
|  |  |  |  |  |  |
|  |  |  |  |  |  |
|  |  |  |  |  |  |
|  |  |  |  |  |  |
|  |  |  |  |  |  |
|  |  |  |  |  |  |
|  |  |  |  |  |  |
|  | TGG2 | AT5G25980.2 | BraC01g022910.1 |  |  |
|  | TGG4 | AT1G47600.1 | BraC05g019280.1/BraC05g019300.1/BraC05g019980.1/BraC08g002750.1/BraC08g002770.1/BraC08g002780.1/BraC08g002790.1/BraC08g002800.1/BraC08g002810.1 | BraA05t20900Z/BraA05t20860Z/BraA05t20859Z | BraA08g002600.3C |
|  |  |  |  |  |  |
|  |  |  |  |  |  |
|  | TGG5 | AT1G51470.1 |  |  |  |
|  | ESP | AT1G54040.2 | BraC05g017180.1 | BraA06t23274Z | BraA05g016540.3C |
|  |  |  | BraC06g000870.1 | BraA06t23299Z | BraA06g000920.3C |
|  |  |  | BraC06g000940.1 | BraA05t20667Z | BraA06g000990.3C |
|  |  |  | BraC06g000890.1 | BraA06t27588Z | BraA06g000940.3C |
|  |  |  | BraC06g050270.1 |  | BraA06g043050.3C |
|  | NSP1 | AT3G16400.1 | BraC01g040520.1/BraC05g037200.1/BraC05g037220.1/BraC01g040610.1/BraC05g037250.1/BraC05g037260.1/BraC08g002480.1/BraC08g002470.1/BraC08g002500.1/BraC06g002210.1 | BraA01t03844Z/BraA05t22131Z/BraA05t22134Z | BraA01g035980.3C/BraA05g030730.3C/BraA05g030710.3C/BraA05g030760.3C/BraA05g030770.3C/BraA05g030780.3C/BraA06g002240.3C//BraA08g002330.3C/BraA08g002350.3C/BraA08g002340.3C |
|  |  |  |  |  |  |
|  | NSP4 | AT3G16410.1 |  |  |  |
|  | NSP3 | AT3G16390.1 |  |  |  |
|  | NSP2 | AT2G33070.3 | BraC04g025420.1 | BraA04t18008Z | BraA06g002260.3C/ |
|  |  |  | BraC06g002230.1 | BraA06t23421Z | BraA04g023310.3C |
|  | NSP5 | AT5G48180.1 | BraC06g042590.1 | BraA02t08715Z | BraA02g039460.3C |
|  |  |  | BraC02g041720.1 | BraA06t26848Z | BraA06g035760.3C |
|  | PEN2 | AT2G44490.1 | BraC05g004690.1 | BraA05t19440Z | BraA05g004390.3C |
|  |  |  | BraC04g033220.1 | BraA04t18796Z |  |
|  | PEN3 | AT1G59870.1 | BraC07g025410.1 | BraA07t30272Z | BraA07g025410.3C |
|  | CAD1 | AT5G44070.1 | BraC06g050550.1 | BraA06t27618Z | BraA06g043350.3C |
|  |  |  | BraC02g033390.1 | BraA02t07857Z | BraA02g031060.3C |
|  |  |  | BraC09g019650.1 | BraA09t37683Z | BraA09g020220.3C |
|  |  |  | BraC09g019640.1 | BraA09t37684Z | BraA09g020230.3C |

Note：The colored blue genes refer to occur tandem duplication

**Supplementary Table S17**. Positional information for newly annotated TGGs in Chiifu v3.0.

| **Gene name** | **Region** |
| --- | --- |
| BrTGG01 | A02:28527044-28529782 |
| BrTGG02 | A02:28547507-28550830 |
| BrTGG03 | A02:28560753-28563484 |
| BrTGG04 | A02:28717631-28720353 |
| BrTGG05 | A05:11274038-11276875 |
| BrTGG06 | A05:11280921-11283750 |
| BrTGG07 | A05:11287796-11290631 |
| BrTGG08 | A05:11722764-11725594 |
| BrTGG09 | A08:1844628-1847451 |
| BrTGG10 | A08:1870960-1873783 |
| BrTGG11 | A08:1876007-1908176 |
| BrTGG12 | A08:1910184-1913006 |
| BrTGG13 | A08:1919840-1922664 |
| BrTGG14 | A08:9588429-9591142 |
| BrTGG15 | A08:20482703-20485498 |
| BrTGG16 | A09:5821327-5823972 |
| BrTGG17 | A09:27838163-27841029 |
