# Supplementary figures and images for: A chromosome-level reference genome of non-heading Chinese cabbage [Brassica campestris (syn. Brassica rapa) ssp. chinensis]
Source: Hortic Res. 2020 Dec 28;7:212. doi: 10.1038/s41438-020-00449-z (PMC7769993; doi:10.1038/s41438-020-00449-z)

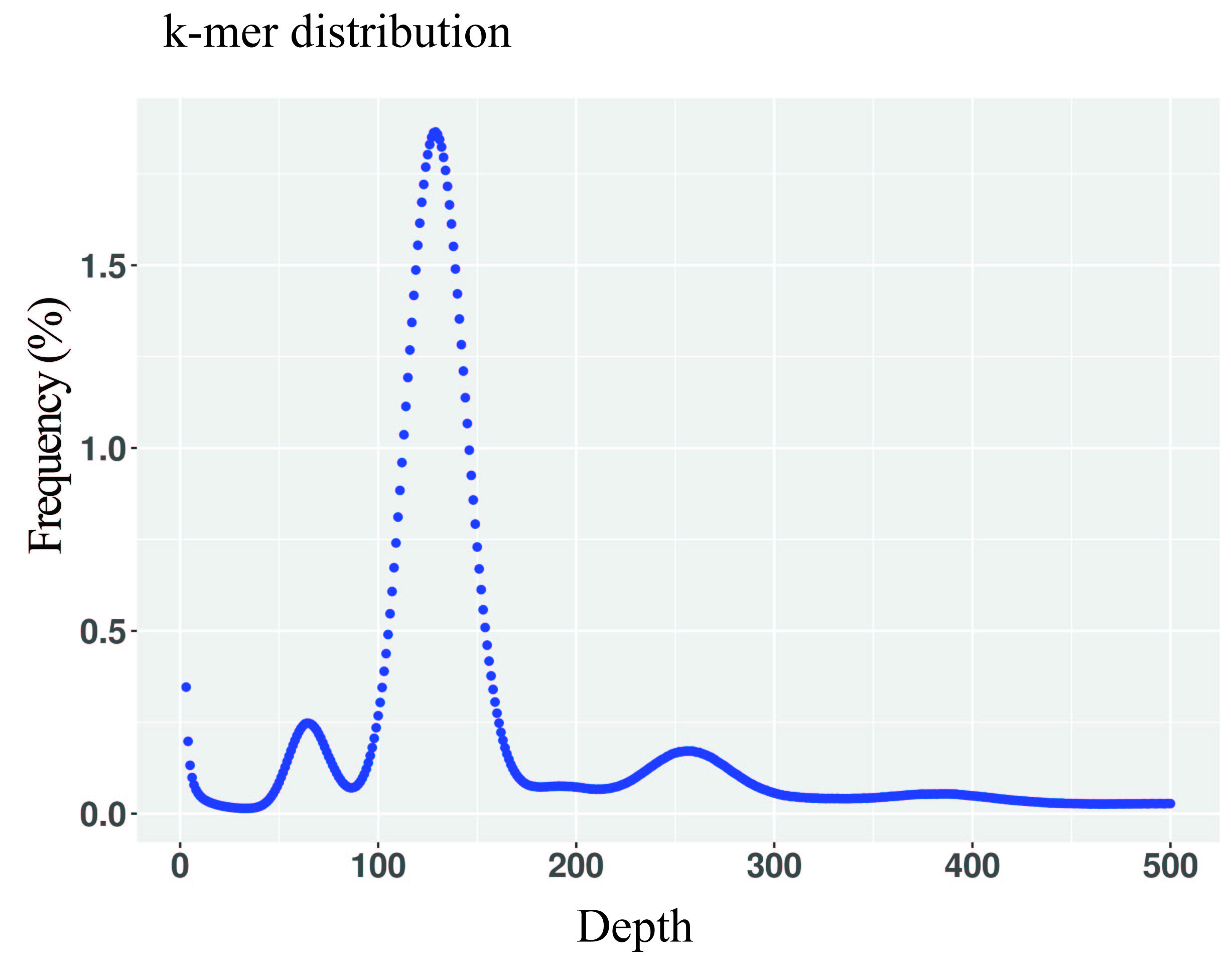

Supplement: Supplementary file 6 — Supplementary Figure S1 [file 41438_2020_449_MOESM6_ESM.jpg]

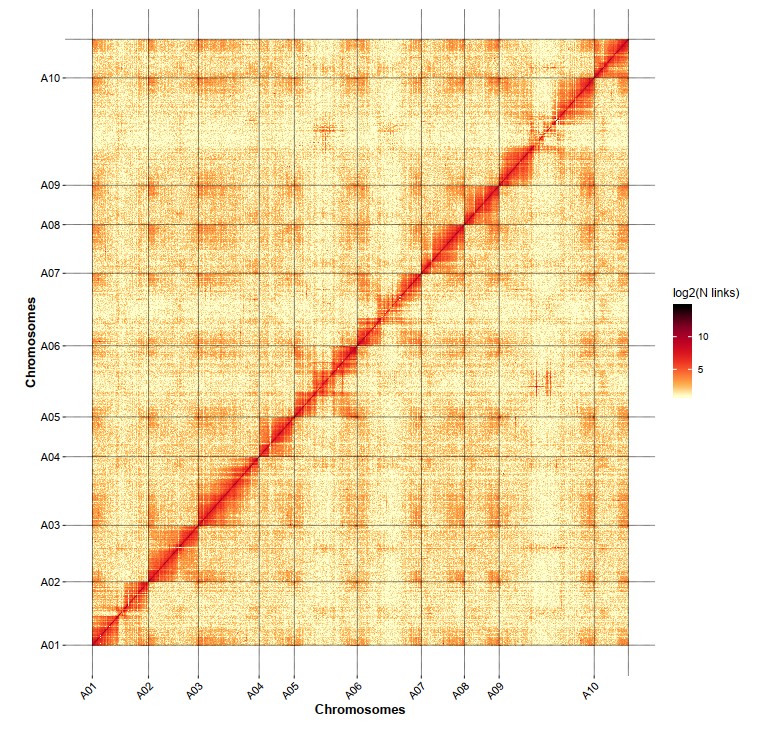

Supplement: Supplementary file 7 — Supplementary Figure S2 [file 41438_2020_449_MOESM7_ESM.jpg]

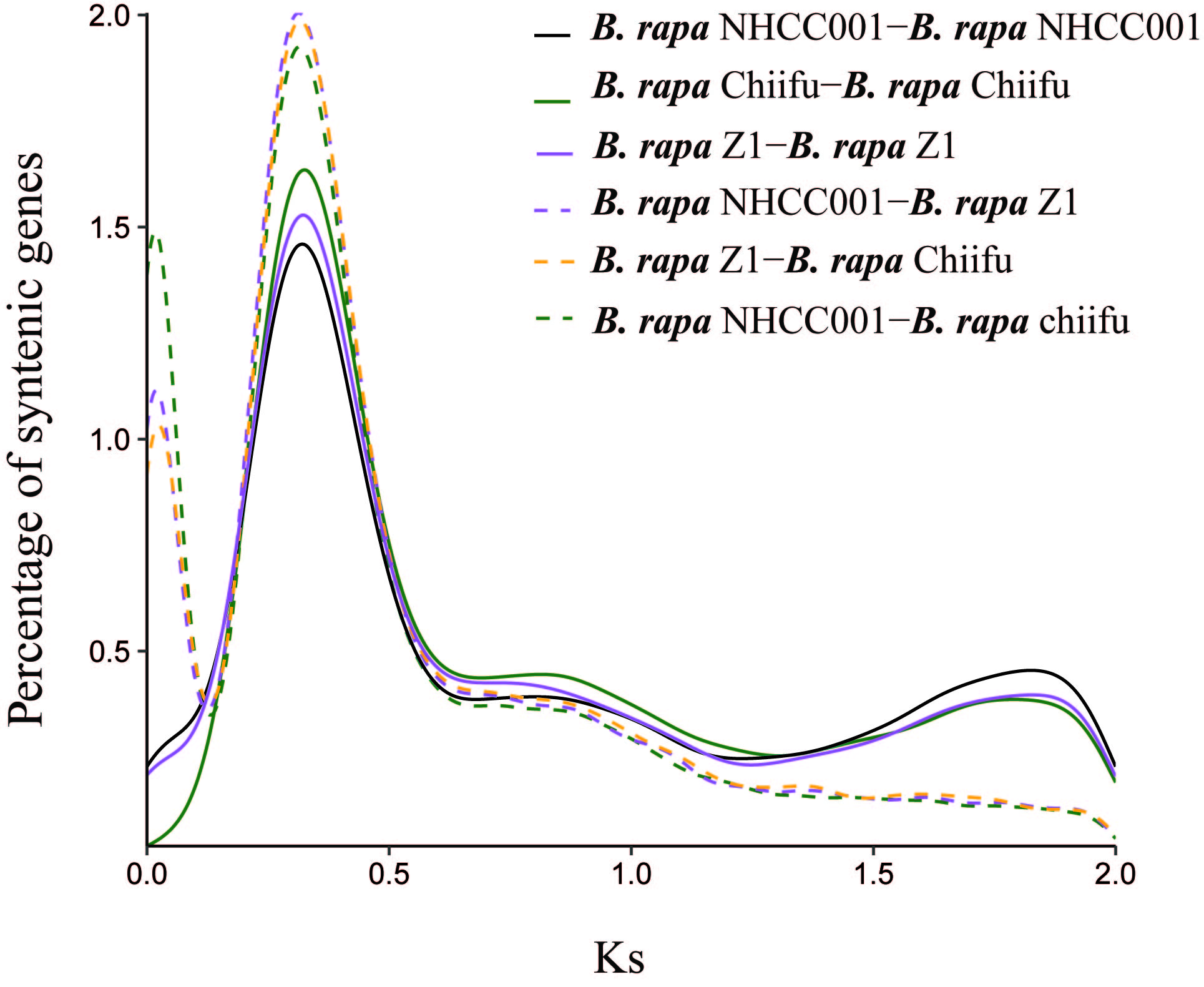

Supplement: Supplementary file 8 — Supplementary Figure S3 [file 41438_2020_449_MOESM8_ESM.jpg]

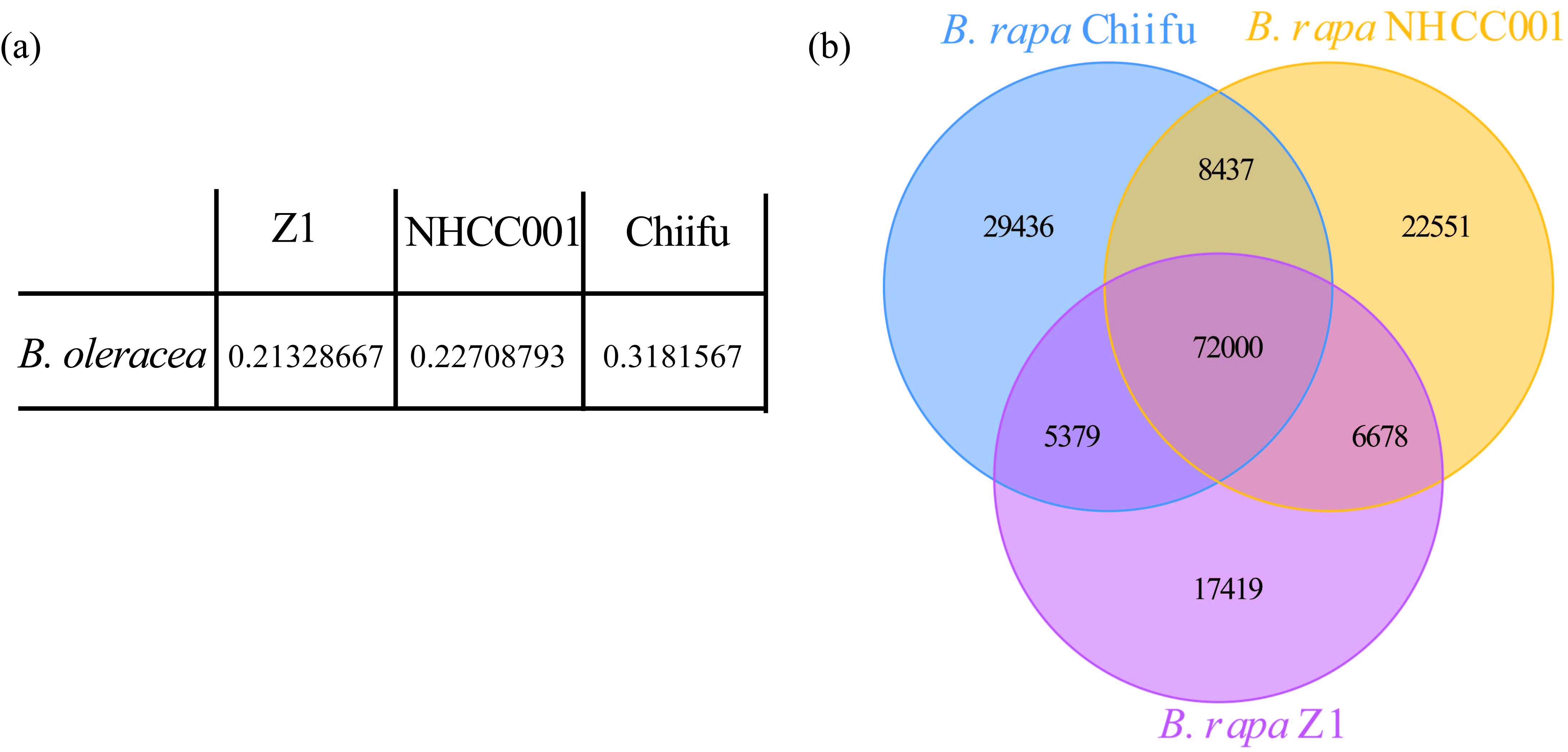

Supplement: Supplementary file 9 — Supplementary Figure S4 [file 41438_2020_449_MOESM9_ESM.jpg]
