# Supplementary material for: A chromosome-level reference genome of non-heading Chinese cabbage [Brassica campestris (syn. Brassica rapa) ssp. chinensis]
Source: Hortic Res. 2020 Dec 28;7:212. doi: 10.1038/s41438-020-00449-z (PMC7769993; doi:10.1038/s41438-020-00449-z)
Supplement: Supplementary file 10 — Supplementry information [file 41438_2020_449_MOESM10_ESM.docx]

**Supplementary Figures**


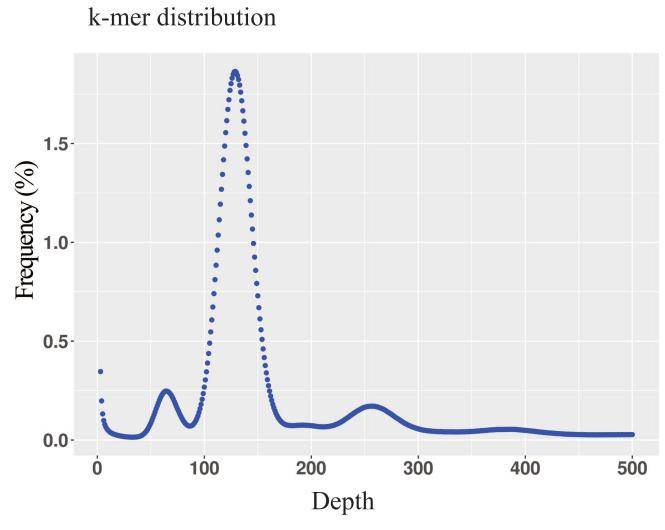


**Supplementary Figure S1**. *k*-mer analysis for estimation of NHCC001 genome size. The peak of the distribution is at approximately 128X coverage, and the genome size was estimated at approximately 477.76 Mb. The small peak at 1/2 the peak depth shows the high heterozygosity rate of the genome.


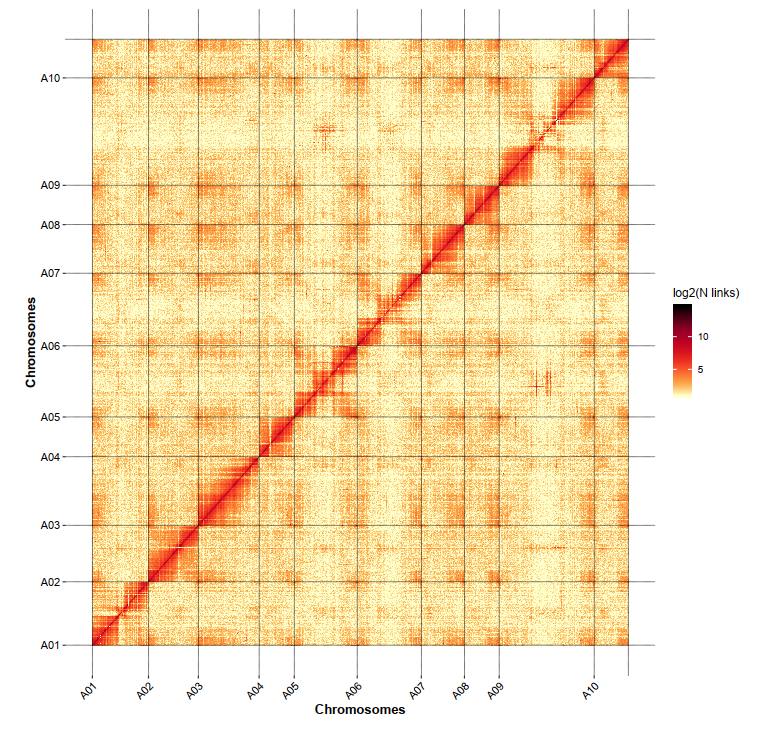


**Supplementary Figure S2** Hi-C interaction heat map for the NHCC001 reference genome showing interactions between the 10 chromosomes.


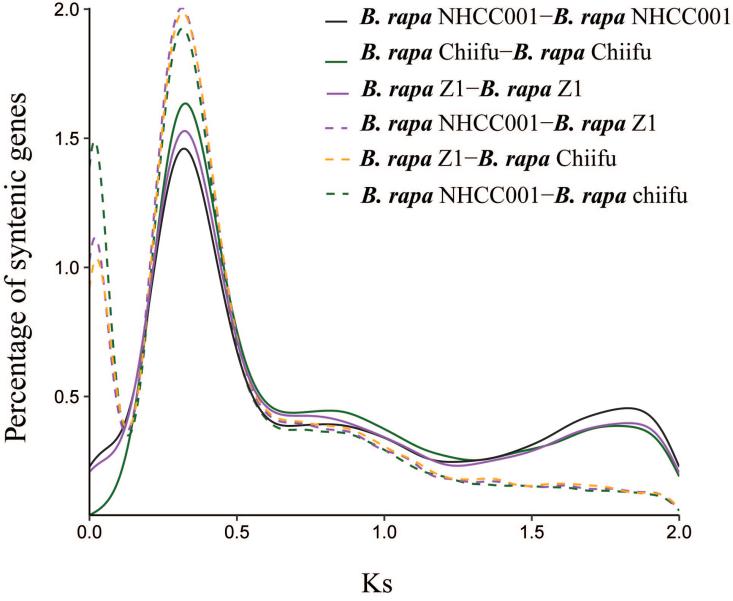


**Supplementary Figure S3** Distributions of synonymous substitution rates (Ks). Ks value distributions between *B. rapa* NHCC001, *B. rapa* Chiifu, and *B. rapa* Z1 are shown.


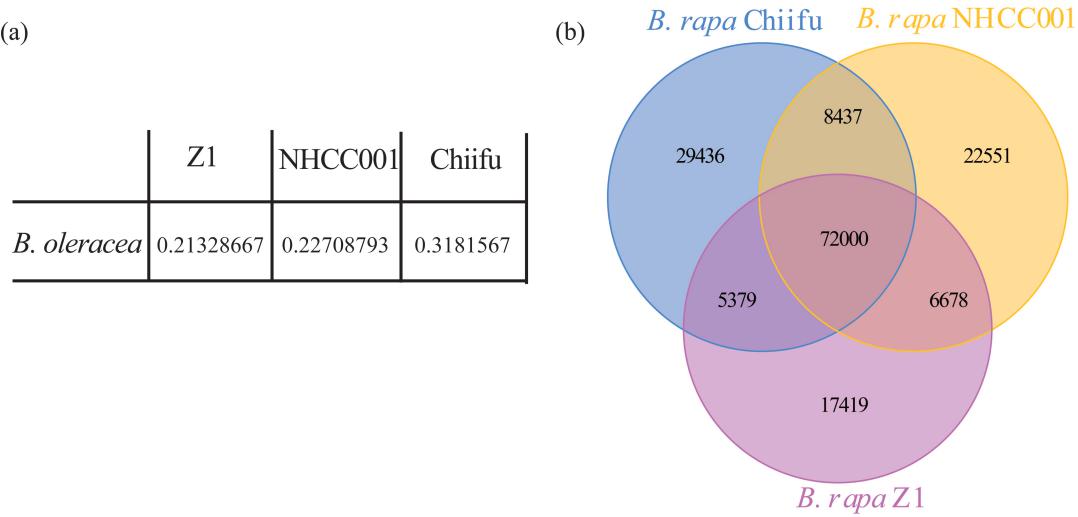


**Supplementary Figure S4** Rates of evolution relative to that of *B. oleracea* and protein mutation sites among the three *B. rapa* genomes.

**Supplementary Tables**

**Supplementary Table S1** Statistics of the ONT and PacBio datasets. Standard metrics were computed using ONT reads (≥2000 bp) and PacBio reads (≥500 bp).

**Supplementary Table S2** Summary of the Hi-C assembly data.

**Supplementary Table S3** Quality assessment of the assembled genome using BUSCO v4.0.6.

**Supplemental Table S4** Error base percentage statistics for the *B. rapa* NHCC001 genome assembly.

**Supplemental Table S5** Genome completeness evaluated based on full-length transcripts.

**Supplementary Table S6** Quality assessment of the assembled genome and gene predictions using RNA-seq data.

**Supplementary Table S7** Summary of the gene functional annotations in the *B. rapa* NHCC001 genome.

**Supplementary Table S8** Statistics for TEs and repeats in the *B. rapa* NHCC001 genome.

**Supplementary Table S9** Statistics for gene families among 14 selected plant genomes.

**Supplementary Table S10** Statistics for SNPs and indels identified between *B. rapa* NHCC001, *B. rapa* Chiifu, and *B. rapa* Z1.

**Supplementary Table S11** Summary of PAV sequences between the *B. rapa* NNHCC001 and *B. rapa* Z1 genomes and between the *B. rapa* NNHCC001 and *B. rapa* Chiifu genomes.

**Supplementary Table S12** Summary of PAV clusters longer than 500 kb between the *B. rapa* NNHCC001 and *B. rapa* Chiifu genomes.

**Supplementary Table S13** Summary of PAV clusters longer than 500 kb between the *B. rapa* NNHCC001 and *B. rapa* Z1 genomes.

**Supplementary Table S14** Leaf adaxial-abaxial patterning genes reported in *A. thaliana* and their homologs identified in *B. rapa* NHCC001, *B. rapa* Chiifu, and *B. rapa* Z1.

**Supplementary Table S15** Ascorbic acid-related genes reported in *A. thaliana* and homologs identified in *B. rapa* NHCC001, *B. rapa* Chiifu, and *B. rapa* Z1.

**Supplementary Table S16** GSL genes reported in *A. thaliana* and homologs identified in *B. rapa* NHCC001, *B. rapa* Chiifu, and *B. rapa* Z1.

**Supplementary Table S17** Positional information for newly annotated TGGs in Chiifu v3.0.

**Supplementary Table S18** Expression levels of 45,158 genes in root and leaf tissues.

**Supplementary Table S19** Detailed sub-genome information for the *B. rapa* NHCC001 genome.

**Supplementary Table S20** Genomic blocks of the Ancestral Crucifer Karyotype (ACK) in the *B. rapa* NHCC001 genome.

**Supplementary Table S21** List and functional annotations of NHCC001- and Chiifu-specific genes in the NHCC001 and Chiifu genomes.
